# On the Security and Efficiency of TLS 1.3 Handshake with Hybrid Key Exchange from CPA-Secure KEMs

**DOI:** 10.3390/e27121242

**Published:** 2025-12-08

**Authors:** Jinrong Chen, Wei Peng, Yi Wang, Yutong Bian

**Affiliations:** College of Computer Science and Technology, National University of Defense Technology, Changsha 410073, China; jinrongchen@nudt.edu.cn (J.C.); yutongbian@nudt.edu.cn (Y.B.)

**Keywords:** TLS 1.3, hybrid key exchange, CPA-secure KEMs, post-quantum cryptography, security proof, performance evaluation, IND-CPA

## Abstract

TLS 1.3 is a crucial protocol for securing modern internet communications. To facilitate a smooth transition to post-quantum security, hybrid key exchange, which combines classical key exchange algorithms with post-quantum key encapsulation mechanisms (KEMs), is proposed to enhance the security of the current TLS 1.3 handshake. However, existing drafts and implementations of hybrid key exchange for TLS 1.3 primarily rely on CCA-secure KEMs (i.e., secure against chosen-ciphertext attacks) based on the Fujisaki-Okamoto (FO) transform. The re-encryption step in their decapsulation algorithms not only introduces additional performance overhead but also raises the risk of side-channel attacks. Although Huguenin-Dumittan and Vaudenay (Eurocrypt 2022) and Zhou et al. (Asiacrypt 2024) demonstrated that the weaker CPA-secure KEMs (i.e., secure against chosen-plaintext attacks) suffice for constructing a secure TLS 1.3 handshake, their analyses were limited to single-KEM settings and did not consider the hybrid key exchange scenario. This work challenges the necessity of CCA security by proving that CPA-secure KEMs are sufficient for the TLS 1.3 handshake even in the hybrid key exchange setting. We provide the first formal security proofs for this claim, covering both the classical random oracle model (ROM) and the quantum random oracle model (QROM), thereby ensuring security against quantum adversaries. To validate the practical benefits, we conduct an extensive performance evaluation based on the latest OpenSSL implementation. Our results show that using CPA-secure KEMs yields up to 44.8% performance improvement at the key exchange layer and up to approximately 9% acceleration for the full TLS 1.3 handshake. Beyond performance gains, this approach reduces the codebase’s attack surface by eliminating the re-encryption step, thereby mitigating a class of side-channel vulnerabilities. Our work positions CPA-secure KEMs as a secure, efficient, and practical alternative for standardizing and deploying post-quantum TLS 1.3 even with hybrid key exchange.

## 1. Introduction

The rapid advancement of quantum computing poses a severe threat to the security of widely deployed public-key infrastructure [1]. Shor’s algorithm [2] signifies that once quantum computing technology matures, public-key cryptosystems based on traditional hardness assumptions such as the discrete logarithm [3] and integer factorization [4] problems will become insecure. TLS 1.3 [5], the predominant protocol for securing internet communication, relies on (elliptic curve) Diffie-Hellman ((EC)DH) key exchange [3,6], which is vulnerable to Shor’s algorithm. Consequently, the existing TLS 1.3 handshake protocol is not secure against adversaries with a quantum computer. To counter “harvest-now-decrypt-later” attacks, there is significant research interest in developing post-quantum TLS 1.3 handshake protocols that run on classical computers yet remain secure against quantum-capable adversaries [7,8,9,10,11,12]. A prevalent approach within the community is to replace the current (EC)DH key exchange with a post-quantum key encapsulation mechanism (KEM), which is considered an effective strategy for achieving a post-quantum TLS 1.3 handshake.

**Figure 1:**
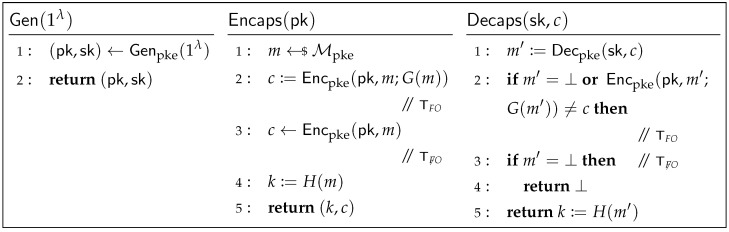
The FO transform $\mathrm{KEM}:=T_{FO}\left[\mathrm{PKE},H,G\right]$ and its counterpart without re-encryption $\mathrm{KEM}:=T_{\text{F̸O}}\left[\mathrm{PKE},H\right]$. $G:M_{\mathrm{pke}}\to R_{\mathrm{pke}}$ and $H:M_{\mathrm{pke}}\to K_{\mathrm{kem}}$ are hash functions, $M_{\mathrm{pke}}$ is the message space of $\mathrm{PKE}=({\mathrm{Gen}}_{\mathrm{pke}},{\mathrm{Enc}}_{\mathrm{pke}},{\mathrm{Dec}}_{\mathrm{pke}})$, $R_{\mathrm{pke}}$ is the randomness space of ${\mathrm{Enc}}_{\mathrm{pke}}$, ${\mathrm{Enc}}_{\mathrm{pke}}\left(\mathrm{pk},m;G\left(m\right)\right)$ denotes explicitly setting the randomness used by ${\mathrm{Enc}}_{\mathrm{pke}}\left(\mathrm{pk},m\right)$ to G(m), and $K_{\mathrm{kem}}$ is the key space of KEM. It is worth noting that the FO transform has other variants (e.g., where the key *k* of KEM is set as k:=H(m,c) instead of H(m)); more information is available in [15].

For the past, it was widely believed that the post-quantum KEM replacing (EC)DH should achieve IND-CCA (i.e., indistinguishable against chosen-ciphertext attacks) security [7,8]. In practice, for the post-quantum KEM recently standardized by the US National Institute of Standards and Technology (NIST), MLKEM [13], and the upcoming HQC candidate [14], both of them rely on the Fujisaki-Okamoto (FO) transform variant [15], which constructs the IND-CCA secure KEMs from the IND-CPA (i.e., indistinguishable against chosen-plaintext attacks) secure public-key encryption (PKE), as shown in Figure 1.

From Figure 1, we can see that KEM’s decapsulation algorithm Decaps requires re-encryption of the decrypted message $m^{\prime }$ (i.e., line 2 in Figure 1), but this process not only introduces additional computational overhead but also increases the risk of side-channel attacks [16,17]. At Eurocrypt 2022, Huguenin-Dumittan and Vaudenay [10] were the first to demonstrate that a KEM with the weaker OW-CPA (i.e., one-way against chosen-plaintext attacks) security is sufficient for constructing a secure TLS 1.3 handshake in the random oracle model (ROM) [18]. This result was subsequently refined by Zhou et al. [11] at Asiacrypt 2024, who presented tighter security proofs in the ROM based on both OW-CPA and IND-CPA secure KEMs and, crucially, extended the results to the quantum random oracle model (QROM) [19], which further confirms that CPA-secure post-quantum KEMs are adequate for building secure post-quantum TLS 1.3 handshakes. As evident from the construction of CPA-secure KEMs without re-encryption [20] (see Figure 1), the elimination of re-encryption in the decapsulation algorithm reduces computational cost and mitigates the aforementioned side-channel risks.

**Figure 2:**
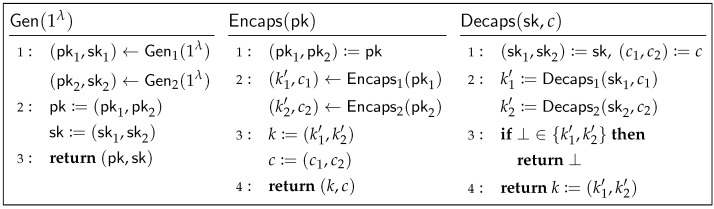
$\mathrm{KEM}:=\mathrm{Comb}[{\mathrm{KEM}}_{1},{\mathrm{KEM}}_{2}]$. Summarized from [21,22].

However, due to ongoing scrutiny of post-quantum KEM security and compatibility concerns, hybrid key exchange schemes, which combine post-quantum KEMs with well-established classical key exchange (e.g., (EC)DH), represent a pragmatic strategy for a smooth transition to post-quantum TLS 1.3. Note that the IETF TLS Working Group is standardizing hybrid key exchange for TLS 1.3 [21,22], with a representative structure depicted in Figure 2 (where (EC)DH is regarded as a KEM as described in [10]).

This hybrid approach essentially employs a simple “concatenation” method, offering the advantage of not requiring additional cryptographic primitives or significant extra computation. It is important to note that the works of Huguenin-Dumittan [10] and Vaudenay and Zhou et al. [11] focus solely on the security of TLS 1.3 using a *single* KEM and do not address the hybrid setting. Critically, the IND-CPA security of individual KEM components does not automatically guarantee the IND-CPA security of the combined KEM. Considering Figure 2, if ${\mathrm{KEM}}_{2}$ is not IND-CPA secure, there exists an adversary A could effectively distinguish whether $k_{2}^{\prime }$ in $\left(k_{2}^{\prime },c_{2}\right)$ is the real key or a random key from $K_{{\mathrm{kem}}_{2}}$. Then, A could break the IND-CPA security of the combined KEM by using $\left(k_{2}^{\prime },c_{2}\right)$ from the challenge key and ciphertext $k=(k_{1}^{\prime },k_{2}^{\prime })$ and $c=(c_{1},c_{2})$. Therefore, whether CPA-secure KEMs remain sufficient for constructing a secure TLS 1.3 handshake in the context of hybrid key exchange remains an open problem.

### 1.1. Our Contributions

In this work, we presents the first security analysis of the TLS 1.3 handshake using CPA-secure KEMs within hybrid key exchange setting. Specifically, our contributions are as follows:

1. We provide the first security proofs for the TLS 1.3 handshake based on CPA-secure KEMs in a hybrid key exchange scenario, both in the ROM and QROM. This formally establishes that CPA-secure KEMs are indeed sufficient for constructing secure post-quantum TLS 1.3 handshakes even when combined with classical key exchange.
2. We implement and evaluate the performance of the hybrid schemes specified in the IETF draft [21], i.e., X25519MLKEM768, SecP256r1MLKEM768, and SecP384-MLKEM1024, using the latest OpenSSL (https://github.com/openssl/openssl/releases/download/openssl-3.6.0/openssl-3.6.0.tar.gz accessed on 23 October 2025). Our experimental results compare the efficiency when the underlying base KEM provides IND-CCA security versus IND-CPA security, and demonstrate that at the key exchange layer, using a CPA-secure KEM can yield a performance improvement of up to approximately 44.8%.
3. Finally, we benchmark the overall TLS 1.3 handshake performance in the hybrid setting for both IND-CCA and IND-CPA secure underlying base KEMs. The experimental results show that at the full protocol level, the efficiency gain from using CPA-secure KEMs is more modest, not exceeding 9%. Nonetheless, employing CPA-secure KEMs still reduces the protocol’s vulnerability to side-channel attacks. Furthermore, eliminating the need for extra hash function calls (e.g., in re-encryption) can potentially save “hundreds of thousands or even millions of dollars a year” in operational costs [23].

In summary, this work provides a comprehensive analysis of both the security and efficiency of the TLS 1.3 handshake with hybrid key exchange from CPA-secure KEMs. Our findings offer additional options and support for the ongoing standardization of post-quantum TLS 1.3.

### 1.2. Technique Overview

**Figure 3:**
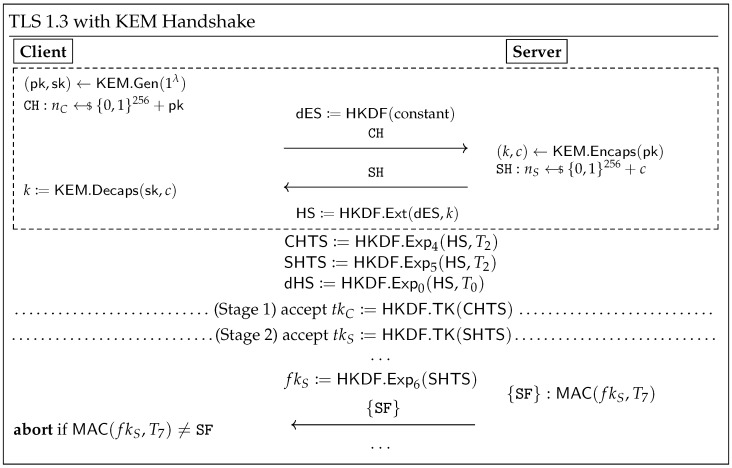
TLS 1.3 handshake with KEM, adapted from [10]. {·} denotes messages encrypted using $tk_{S}$, and $T_{i}$ is the hash of the transcript up to the *i*-th message. For brevity, ClientHello and ClientKeyShare are merged into CH, while ServerHello and ServerKeyShare are merged into SH. Only some important steps are shown. The remaining stage keys (3-6, omitted) are derived from dHS.

The KEM-based TLS 1.3 handshake protocol is depicted in Figure 3. During the key exchange, the client first computes $\left(\mathrm{pk},\mathrm{sk}\right)\leftarrow \mathrm{KEM}.\mathrm{Gen}\left(1^{\lambda }\right)$ and sends pk to the server (as the client share). Upon receiving pk, the server computes (k,c)←KEM.Encaps(pk) and sends *c* to the client (as the server share). Finally, the client computes k:=KEM.Decaps(sk,c) upon receiving *c*. Both parties then use *k* as the shared secret.

#### 1.2.1. Security Proof of TLS 1.3 Handshake with Hybrid Key Exchange from CPA-Secure KEMs

**Figure 4:**
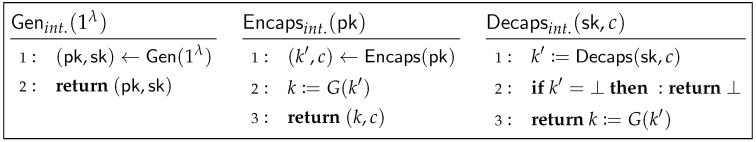
The *intermediate* KEM ${\mathrm{KEM}}_{int.}:=T_{int.}\left[\mathrm{KEM},G\right]$, adapted from [12], where *G* is a key derivation function (corresponding to HKDF.Ext(dES,·) in Figure 3).

Analyzing the security of TLS 1.3 handshake with hybrid key exchange is non-trivial. The protocol comprises numerous cryptographic primitives (e.g., key derivation function HKDF, message authentication code MAC, etc.), intertwined in a manner where their security properties mutually influence each other, collectively forming the complex TLS 1.3 protocol. Fortunately, recent work by Chen et al. [12] introduces a modular analysis framework. They conceptualize the component within the dashed box in Figure 3 as a new KEM, termed the *intermediate* KEM (see Figure 4).

**Figure 5:**
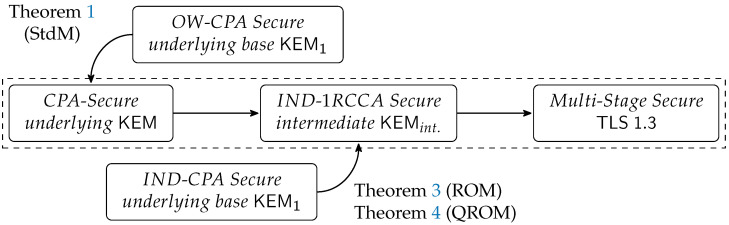
Modular analysis of TLS 1.3 handshake. $\begin{array}{c} A \end{array}\to \begin{array}{c} B \end{array}$ denotes that *A* implies *B* provided the other cryptographic components are secure. Results inside the dashed box were established by Chen et al. [12], while those outside it are contributions of this work. The notes following the theorems indicate the proof model, where StdM denotes the (random oracle independent) standard model.

From this perspective, the client and the server can be viewed as using this *intermediate* KEM to negotiate HS as their “shared secret” following the aforementioned process. Based on the *intermediate* KEM, Chen et al. [12] proposed a modular analysis approach, as illustrated in the section delineated by the dashed box in Figure 5.

Briefly, Chen et al. [12] prove that the OW-CPA or IND-CPA security of the underlying KEM implies the IND-1RCCA security of the *intermediate* KEM, and that IND-1RCCA security of the *intermediate* KEM suffices to guarantee the overall security of the TLS 1.3 handshake. Although Chen et al. [12] focused on single KEM instances, their modular approach can be extended to the hybrid key exchange scenario. When TLS 1.3 employs hybrid key exchange, the underlying KEM in Figure 5 is constructed by combining several *base KEMs* according to the method shown in Figure 2. This modular analysis simplifies the security analysis of the TLS 1.3 handshake, allowing us to concentrate our security focus primarily on the KEM’s security.

Conclusion 1: When one base KEM satisfies OW-CPA security (Section 3.1). The security analysis for the TLS 1.3 handshake in this case is relatively straightforward. We need only ensure that the underlying hybrid KEM (in Figure 5) is OW-CPA secure as a whole. This can be proven by contradiction: if there exists a PPT adversary A that can break the hybrid KEM, we can construct a new PPT adversary B from A that breaks the OW-CPA security of any constituent base KEM. Given that at least one base KEM is OW-CPA secure, such an adversary A cannot exist, thereby proving the OW-CPA security of the hybrid KEM (see Theorem 1). Once the hybrid KEM’s OW-CPA security is established, combining this with Chen et al.’s result [12] proves that the TLS 1.3 handshake based on hybrid key exchange is secure as long as one base KEM is OW-CPA secure (see Theorem 2).
Conclusion 2: When one base KEM satisfies IND-CPA security (Section 3.2). The security analysis here is slightly more complex. As discussed in the paragraph following Figure 2, the IND-CPA security of a single base KEM does *not* necessarily imply the IND-CPA security of the hybrid KEM. Therefore, we cannot rely on a simple analysis of the hybrid KEM’s security as in the OW-CPA case. Fortunately, we can shift our focus to the IND-1RCCA security of the *intermediate* KEM. If we can prove that the IND-CPA security of one base KEM implies the IND-1RCCA security of the *intermediate* KEM, then, combined with Chen et al.’s results [12], we can conclude that the TLS 1.3 handshake with hybrid key exchange is secure (Theorem 5). Depending on the adversary’s capabilities, we provide security proofs in both the ROM (Theorem 3) and the QROM (Theorem 4).
Case 1: The proof of the KEM in the ROM (Section 3.2.1). The key to reducing the IND-1RCCA security of the *intermediate* KEM to the IND-CPA security of a base KEM lies in simulating the decapsulation oracle in the IND-1RCCA security game without access to the private key sk. We consider three cases based on the value $k^{\prime }:=\mathrm{Decaps}\left(\mathrm{sk},c\right)$ computed by the decapsulation oracle:
  1. When $k^{\prime }=k^{*}$ (where $G(k^{*})$ is the challenge key), the actual decapsulation result $G(k^{\prime })$ must equal the challenge key, so the oracle returns test.
  2. When $k^{\prime }=⊥$, the oracle returns ⊥, consistent with the actual decapsulation algorithm.
  3. For other values of $k^{\prime }$, the oracle must query the random oracle *G* on $k^{\prime }$ to determine the return value. If the adversary has not queried $k^{\prime }$ itself, the return value is random and independent from the adversary’s view. Consequently, in this case, the oracle can use a *pre-selected* random value to determine its return value. When handling the adversary’s random oracle queries, the reduction can randomly select one query (or with some probability, none) to respond with this pre-selected value. This achieves the goal of determining the oracle’s return value without needing to use sk to compute $k^{\prime }$.

Thus, the reduction algorithm can guess which of the three cases applies for the single decapsulation query (succeeding with probability 1/3), enabling it to simulate the IND-1RCCA decapsulation oracle effectively without using sk to compute $k^{\prime }$.

Case 2: The proof of the KEM in the QROM (Section 3.2.2). When considering the quantum-capable adversaries, we should extend the security proof to the QROM. The core challenge remains simulating the IND-1RCCA decapsulation oracle without sk. We can still employ random guessing for the decapsulation result: if $k^{\prime }=k^{*}$ is guessed, the oracle returns test; if $k^{\prime }=⊥$ is guessed, it returns ⊥. The key difference arises in the remaining case. Due to fundamental quantum principles (specifically, the no-cloning principle), we cannot simply record the adversary’s “quantum” queries without significantly disturbing its state, as was possible in the ROM. However, we can utilize the *singular-classical-query measure-and-reprogram lemma* [24] to deal with this problem. This technique employs a measure-and-reprogram approach for the random oracle, allowing for simulation of the decapsulation oracle without sk and enabling the security proof in the QROM.

#### 1.2.2. Efficiency Analysis of TLS 1.3 Handshake with Hybrid Key Exchange from CPA-Secure KEMs

We conduct an experimental performance analysis of both the hybrid key exchange itself and the full TLS 1.3 handshake with hybrid key exchange, based on OpenSSL (https://openssl-library.org/ accessed on 23 October 2025), a widely-used cryptography library. The latest OpenSSL version supports three hybrid key exchange methods that are specified in the IETF draft [21]: X25519MLKEM768, SecP256r1MLKEM768, and SecP384r1MLKEM1024. The MLKEM implementation follows NIST FIPS 203 standard [13] and provides IND-CCA security.

**Figure 6:**
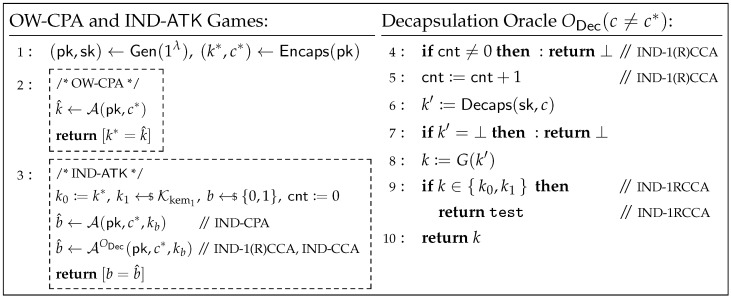
Security games for OW-CPA and IND-ATK (where ATK∈{CPA,1RCCA,1CCA,CCA}) security of KEM=(Gen,Encaps,Decaps).

Experimental Analysis at the Key Exchange Level (Section 4.1). We modify the OpenSSL source code related to MLKEM, specifically altering its encapsulation and decapsulation algorithms according to ([20], Figure 6) (denoted as $T_{\text{F̸O}}$ in this work and shown in Figure 1) to effectively reduce its security to IND-CPA. After compiling them, we use the openssl speed tool to obtain performance data for the hybrid key exchange, allowing us to analyze the performance improvement gained by removing re-encryption.
Experimental Analysis at the Protocol Level (Section 4.2). For protocol-level testing of TLS 1.3, we simulate the handshake between a client and a server locally using two terminals, thereby eliminating external network latency and focusing solely on protocol efficiency. Specifically, we start one terminal to act as the server (after generating its certificate) using the openssl s_server tool. Then, we use another terminal with the openssl s_time tool, acting as the client, to measure the handshake time with the server. However, there are two practical issues: first, openssl s_time does not allow for specification of the key exchange algorithm for the handshake; second, the default list of key exchange algorithms supported by openssl s_time does not include all the hybrid schemes.

To address the first issue, we can use the -groups option of openssl s_server to restrict the server to support only one specific key exchange method, thereby forcing openssl s_time to use that method. For the second issue, we modify the OpenSSL configuration file (openssl.cnf) to include all the hybrid schemes in OpenSSL’s default key exchange list. With this setup, we establish a local test environment for TLS 1.3 using different hybrid key exchange schemes, enabling further analysis of the performance benefits of using weaker-security KEMs at the protocol level.

### 1.3. Related Work

Since the formal standardization of TLS 1.3 [5], numerous researchers have investigated its security [25,26,27]. Regarding the handshake protocol specifically, Dowling et al. [8] extended the analysis of earlier TLS 1.3 drafts [28,29,30,31,32]. They formally defined the multi-stage key exchange model to comprehensively capture the properties of keys derived in different stages of the TLS 1.3 handshake and provided a formal security proof in the standard model based on the PRF-ODH assumption [33]. As research progressed, many began exploring the integration of KEMs into TLS 1.3 to enhance its security [7,8,9,10,11,12]. For KEMs intended to replace (EC)DH key exchange, much prior work [7,8] assumed that IND-CCA security was necessary. However, Huguenin-Dumittan and Vaudenay [10] were the first to prove, in the ROM, that OW-CPA secure KEMs are sufficient for the TLS 1.3 handshake’s security, concurrently showing that the CDH assumption [3] suffices for proving the security of the existing TLS 1.3. Zhou et al. [11] subsequently improved the security analyses by Huguenin-Dumittan and Vaudenay [10] in the ROM and, crucially, extended the result to the QROM, demonstrating that CPA-secure KEMs also suffice for post-quantum TLS 1.3 security.

Note that the aforementioned studies primarily focus on TLS 1.3 security with a *single* KEM and do not address the impact of *hybrid* key exchange. In practice, however, many institutions like Google and Cloudflare are gradually deploying hybrid key exchange in TLS 1.3 (https://www.intelligentliving.co/quantum-hybrid-tls-ml-kem-browser/ accessed on 23 October 2025), combining classical key exchange with post-quantum KEMs, to facilitate a smooth transition from classical to post-quantum security. This trend is accompanied by several IETF drafts proposing methods for hybrid key exchange in TLS [21,22]. Researchers have also extensively studied various hybrid key exchange methods [34,35,36], and recent work by Liu et al. [37] unified several primary approaches. Nevertheless, these studies did not specifically consider the security implications of using CPA-secure KEMs within the hybrid key exchange framework for the TLS 1.3 handshake.

Regarding benchmarking the post-quantum TLS 1.3 handshake with hybrid key exchange, Zheng et al. [38] provided a detailed analysis of various hybrid schemes based on ML-KEM. However, the ML-KEM they considered provided the stronger IND-1CCA security, not IND-CPA security. Benčina et al. [20] investigated hybrid key exchange using CPA-secure KEMs for the SSH protocol. They analyzed the performance of hybrid key exchange combining X25519 with both CCA-secure and CPA-secure variants of ML-KEM and Streamlined NTRU Prime, as well as the overall SSH protocol performance. However, their findings are not directly applicable to the TLS 1.3 handshake with hybrid key exchange.

### 1.4. Paper Structure

We review necessary preliminaries in Section 2. Section 3 provides the security proofs for TLS 1.3 handshake with hybrid key exchange from CPA-secure KEMs. Section 4 reports our experimental results for both the key exchange level and the protocol level under hybrid key exchange conditions. Finally, Section 5 concludes the paper.

## 2. Preliminaries

This section reviews some cryptographic primitives and relevant lemmas.

### 2.1. Notation

Let H:X→Y denote the function with domain X and codomain Y, and $\Omega_{H}$ be the set of all such functions. For a set S, its size is denoted by |S|, and sampling an element *s* uniformly at random from S is written as $s\leftarrow_{\$}S$. For a probabilistic (resp. deterministic) algorithm *A* with input *x*, we denote its output as *y* by y←A(x) (resp. y:=A(x)). An oracle algorithm *A* with classical (resp. quantum) access to an oracle *H* is denoted by $A^{H}$ (resp. $A^{|H\rangle }$). The symbol [x=y] represents an indicator function that equals 1 if x=y and 0 otherwise. The security parameter is denoted by λ, and probabilistic polynomial time is abbreviated as PPT.

### 2.2. Cryptographic Primitives

**Definition** **1** (Key Encapsulation Mechanism, KEM)**.** *A Key Encapsulation Mechanism (KEM) KEM consists of three PPT algorithms, parameterized by a security parameter λ, a key space K, and a ciphertext space C, where: (1) The key generation algorithm Gen is a probabilistic algorithm that takes $1^{\lambda }$ as input and outputs a public key/secret key pair (pk,sk); (2) The encapsulation algorithm Encaps is a probabilistic algorithm that takes pk as input and outputs a key-ciphertext pair (k,c)∈K×C; (3) The decapsulation algorithm Decaps is a deterministic algorithm that takes (sk,c) as input and outputs k∈K∪{⊥}. We say KEM=(Gen,Encaps,Decaps) is defined over (K,C).*

*The correctness requirement for KEM is that for all $\left(\mathrm{pk},\mathrm{sk}\right)\leftarrow \mathrm{Gen}\left(1^{\lambda }\right)$ and all (k,c)←Encaps(pk), it holds that Decaps(sk,c)=k. If ⊥∉K, KEM is said to have explicit rejection; if* ⊥ *is a random value in K, KEM is said to have implicit rejection.*

**Definition** **2** (Security of KEM)**.** *The OW-CPA security and IND-ATK security (where ATK∈{CPA,1RCCA,1CCA,CCA}) of a KEM KEM=(Gen,Encaps,Decaps) defined over (K,C) are defined through a game played between a challenger and an adversary A, as shown in Figure 6.*

We define the advantage of A against the OW-CPA security of KEM as ${\mathrm{Adv}}_{\mathrm{KEM}}^{\text{OW-CPA}}\left(A\right):=\mathrm{Pr}\left[k^{*}=\hat{k}\right]$, and the advantage against the IND-ATK security of KEM as ${\mathrm{Adv}}_{\mathrm{KEM}}^{\text{IND-ATK}}\left(A\right):=\left|\mathrm{Pr}[b=\hat{b}]-1/2\right|$. If for any PPT adversary A, ${\mathrm{Adv}}_{\mathrm{KEM}}^{\text{OW-CPA}}\left(A\right)$ (or ${\mathrm{Adv}}_{\mathrm{KEM}}^{\text{IND-ATK}}\left(A\right)$) is negligible, then KEM is said to be OW-CPA secure (or IND-ATK secure, respectively). The values $k^{*},k_{0},k_{1}$ sent to A are referred to as the challenge keys, and c is referred to as the challenge ciphertext. Sometimes, to distinguish between $k_{0}$ and $k_{1}$, we also call $k_{0}$ the real key and $k_{1}$ the random key.

### 2.3. (Quantum) Random Oracle Model

The random oracle model (ROM), introduced by Bellare and Rogaway [18], is an important cryptographic model that treats hash functions as ideal public random oracles. In this model, an adversary can only obtain the value of a hash function H(x) by querying the random oracle *H* on *x*; otherwise, H(x) remains independent and random from the adversary’s perspective.

The quantum random oracle Model (QROM) [19] extends the ROM by allowing adversaries to query the random oracle in quantum superposition states. In QROM, the random oracle *H* is modeled as a unitary operator $U_{H}:|x,y\rangle \mapsto |x,y⊕H(x)\rangle$, where the quantum registers containing *x* and *y* are referred to as the input and output registers (of this random oracle *H* query), respectively. This model is crucial for analyzing the security of cryptographic schemes against quantum-capable adversaries, as it captures the adversary’s quantum computational power. For further background on quantum computing, readers may refer to [39].

### 2.4. Important Lemmas

**Lemma** **1** (Double-sided one-way to hiding, DS-O2H ([40] Lemma 5), adapted)**.** Let G,H:X→Y be random functions such that $\forall x\neq x^{*}\in X$, G(x)=H(x), and let z be a random value, where $\left(G,H,x^{*},z\right)$ may have an arbitrary joint distribution. Let $A^{|H\rangle }\left(z\right)$ be an oracle algorithm (not necessarily unitary) with quantum access to random oracle H. Then there exists a double-sided oracle algorithm $B^{|G\rangle ,|H\rangle }\left(z\right)$ with quantum access to both random oracles G and H, such that

$$|\mathrm{Pr}\left[1\leftarrow A^{|G\rangle }\left(z\right)\right]-\mathrm{Pr}\left[1\leftarrow A^{|H\rangle }\left(z\right)\right]|\leq 2\sqrt{\mathrm{Pr}[x^{*}=\hat{x}:\hat{x}\leftarrow B^{|G\rangle ,|H\rangle }\left(z\right)]}\;.$$

**Lemma** **2** (Search in double-sided oracle ([24] Lemma 2.3), adapted)**.** In Lemma 1, if $X=X_{1}\times X_{2}$, $x^{*}=\left(x_{1},x_{2}\right)\in X_{1}\times X_{2}$, and $x_{1}\in X_{1}$ is independent of G (or H) and z, then

$$\mathrm{Pr}\left[x^{*}=\hat{x}:\hat{x}\leftarrow B^{|G\rangle ,|H\rangle }\left(z\right)\right]\leq q_{G}^{2}/|X_{1}|\;\left(or\;q_{H}^{2}/|X_{1}|\right)\;,$$

*where $q_{G}$ (or $q_{H}$) is the bounded number of G (or H) random oracle queries made by $A^{|G\rangle }\left(z\right)$ (or $A^{|H\rangle }\left(z\right)$).*

**Lemma** **3** (Singular-classical-query measure-and-reprogram lemma ([24] Lemma 3.1), adapted)**.** Let $A^{|H\rangle }$ be a quantum oracle algorithm making at most q (quantum) queries to the random oracle H:X→Y, and outputting some classical x∈X and a (possibly quantum) output z. In particular, the input state of the $i^{*}$-th query of $A^{|H\rangle }$ is |x〉, where $i^{*}\in \left\{1,\ldots ,q\right\}$, and |x〉 is a classical state identical to the x output by $A^{|H\rangle }$.

Let $S^{A}\left(\Theta \right)$ be a quantum oracle algorithm that picks a random pair $\left(i,b_{0}\right)\leftarrow_{\$}\left(\left\{1,\ldots ,q\right\}\setminus \left\{i^{*}\right\}\times \left\{0,1\right\}\right)\cup \left\{\left(q+1,0\right)\right\}$, runs $A^{|H_{i}^{i^{*}}\rangle }$ to output z, where $H_{i}^{i^{*}}$ is an oracle that returns Θ for the $i^{*}$-th H query of A, measures the input of the i-th H query of A to obtain x, uses H to answer the l-th H query of A (for $l<(i+b_{0})$ and $l\neq i^{*}$), and uses $H_{x\Theta }$ (where $H_{x\Theta }\left(x\right)=\Theta$ and $H_{x\Theta }\left(x^{\prime }\right)=H\left(x^{\prime }\right)$ for all $x^{\prime }\neq x$) to answer the l-th H query of A (for $l\geq (i+b_{0})$ and $l\neq i^{*}$).

Let $S_{1}^{A}\left(\Theta \right)$ be a quantum oracle algorithm that picks a random pair $\left(j,b_{1}\right)\in \left(\left\{i^{*}+1,\ldots ,q\right\}\times \left\{0,1\right\}\right)\cup \left\{\left(q+1,0\right)\right\}$, runs $A^{|H_{j}\rangle }$ to output z, where $H_{j}$ is an oracle that measures the input of the j-th H query of A to obtain x, uses H to answer the l-th oracle query of A (for $l<(j+b_{1})$), and uses $H_{x\Theta }$ to answer the l-th oracle query of A (for $l\geq (j+b_{1})$).

Then for any $x_{0}\in X$, $i^{*}\in \left\{1,\ldots ,q\right\}$, and any predicate V, we have

$$\begin{array}{cc} \; & \underset{H}{\mathrm{Pr}}\left[x=x_{0}\wedge V\left(x,H\left(x\right),z\right)=1:\left(x,z\right)\leftarrow A^{|H\rangle }\right]\\ \; & \;\leq 2{\left(2q-1\right)}^{2}{\mathrm{Pr}}_{H,\Theta }\left[x=x_{0}\wedge V\left(x,\Theta ,z\right)=1:\left(x,z\right)\leftarrow S^{A}\right]\\ \; & \;+8q^{2}{\mathrm{Pr}}_{H,\Theta }\left[x=x_{0}\wedge V\left(x,\Theta ,z\right)=1:\left(x,z\right)\leftarrow S_{1}^{A}\right]\;, \end{array}$$

where the subscripts in ${\mathrm{Pr}}_{H}$ and ${\mathrm{Pr}}_{H,\Theta }$ indicate that the probability is taken over the random choices of H and Θ. Furthermore, if $V=V_{1}\wedge V_{2}$ and $V_{1}\left(x,y,z\right)=1$ if and only if y is returned in the response to the $i^{*}$-th H-query of A, then

$$\sum_{x_{0}}\underset{H,\Theta }{\mathrm{Pr}}\left[x=x_{0}\wedge V\left(x,\Theta ,z\right)=1:\left(x,z\right)\leftarrow S_{1}^{A}\right]\leq 1/\left|Y\right|\;.$$

**Lemma** **4** (Difference lemma [41])**.** *Let $W_{1},W_{2}$, and Z be events defined in some probability space, and let $\bar{Z}$ denote the complement of Z. If $\mathrm{Pr}\left[W_{1}\wedge \bar{Z}\right]=\mathrm{Pr}\left[W_{2}\wedge \bar{Z}\right]$, then*

$$|\mathrm{Pr}\left[W_{2}\right]-\mathrm{Pr}\left[W_{1}\right]|\;\leq \;\mathrm{Pr}\left[Z\right]\;.$$

## 3. Security of TLS 1.3 Hybrid Handshake

We now prove that CPA-secure KEMs suffice for the TLS 1.3 hybrid handshake, considering both OW-CPA and IND-CPA-secure base KEMs.

### 3.1. When the Underlying Base KEM Is OW-CPA Secure

This case is straightforward. Following the modular analysis (Figure 5), we first prove that the hybrid KEM inherits OW-CPA security if one base KEM is OW-CPA secure (Theorem 1). Then, applying results from [12], we finally establish the security of the TLS 1.3 hybrid handshake from OW-CPA secure KEMs (Theorem 2).

**Theorem** **1** (OW-CPA security of the hybrid KEM)**.** *If ${\mathrm{KEM}}_{1}$ is OW-CPA secure, then $\mathrm{KEM}:=\mathrm{Comb}[{\mathrm{KEM}}_{1},{\mathrm{KEM}}_{2}]$ (as shown in Figure 2) is also OW-CPA secure. Specifically, for any PPT adversary A against KEM, there exists a PPT adversary B against ${\mathrm{KEM}}_{1}$, such that*

$${\mathrm{Adv}}_{\mathrm{KEM}}^{\text{OW-CPA}}\left(A\right)\leq {\mathrm{Adv}}_{{\mathrm{KEM}}_{1}}^{\text{OW-CPA}}\left(B\right)\;.$$

**Proof of Theorem 1.**  The proof constructs an adversary B against ${\mathrm{KEM}}_{1}$ by using adversary A. Given challenge $\left({\mathrm{pk}}_{1},c_{1}\right)$ from its OW-CPA challenger, B generates $\left({\mathrm{pk}}_{2},{\mathrm{sk}}_{2}\right)\leftarrow {\mathrm{Gen}}_{2}\left(1^{\lambda }\right)$ and $\left(k_{2}^{\prime },c_{2}\right)\leftarrow {\mathrm{Encaps}}_{2}\left({\mathrm{pk}}_{2}\right)$, then forms the hybrid challenge $\mathrm{pk}:=({\mathrm{pk}}_{1},{\mathrm{pk}}_{2})$, $c:=(c_{1},c_{2})$ for A. Feeds (pk,c) to A. When A outputs a solution $k=(k_{1},k_{2})$ for KEM, B returns $k_{1}$ as the solution for ${\mathrm{KEM}}_{1}$. The validity of $k_{1}$ follows directly from the winning condition of A. Therefore, we have

$${\mathrm{Adv}}_{\mathrm{KEM}}^{\text{OW-CPA}}\left(A\right)\leq {\mathrm{Adv}}_{{\mathrm{KEM}}_{1}}^{\text{OW-CPA}}\left(B\right)\;.$$

   □

Combining with relevant results from [12], we can obtain the security of TLS 1.3 handshake with hybrid key exchange from OW-CPA secure base KEMs.

**Theorem** **2** (Security of TLS 1.3 handshake with hybrid key exchange from OW-CPA secure KEM)**.** *If the other components in TLS 1.3 handshake are secure (as defined in [12]), then the entire TLS 1.3 handshake with hybrid key exchange is secure, provided that at least one underlying base KEM is OW-CPA secure.*

**Proof of Theorem 2.**  This result follows directly from Theorem 1, combined with Theorems 4.1 and 4.2 of [12] (which show that IND-1RCCA security of the *intermediate* KEM implies a special IND-1CCA-1MAC security), Theorem 5.1 (which establishes that OW-CPA security of the underlying KEM implies IND-1RCCA security of the *intermediate* KEM), and Theorems 6.2 and 6.3 (which prove that IND-1CCA-1MAC security of the *intermediate* KEM implies the multi-stage security of the TLS 1.3 handshake) in the same reference.    □

### 3.2. When the Underlying Base KEM Is IND-CPA Secure

Here, we first prove the IND-1RCCA security of the *intermediate* KEM in the ROM and QROM (Theorems 3 and 4), and then derive the security of the full handshake (Theorem 5).

#### 3.2.1. Security of the *Intermediate* KEM in the ROM

**Theorem** **3** (IND-1RCCA security of the hybrid ${\mathrm{KEM}}_{int.}$ in the ROM)**.** *Let ${\mathrm{KEM}}_{int.}:=T_{int.}\left[\mathrm{Comb}\left[{\mathrm{KEM}}_{1},{\mathrm{KEM}}_{2}\right],G\right]$ (Comb and $T_{int.}$ defined in Figure 2 and Figure 4), with G modeled as a random oracle. If ${\mathrm{KEM}}_{1}$ is IND-CPA secure, then ${\mathrm{KEM}}_{int.}$ is IND-1RCCA secure. Specifically, for any PPT adversary A against ${\mathrm{KEM}}_{int.}$, there exists a PPT adversary B against ${\mathrm{KEM}}_{1}$, such that*

$${\mathrm{Adv}}_{{\mathrm{KEM}}_{int.}}^{\text{IND-1RCCA}}\left(A\right)\leq 6\left(q_{G}+1\right)\cdot {\mathrm{Adv}}_{{\mathrm{KEM}}_{1}}^{\text{IND-CPA}}\left(B\right)+3q_{G}\left(q_{G}+1\right)/|K_{{\mathrm{kem}}_{1}}|\;,$$

*where $q_{G}$ is the number of queries to G made by A, and $K_{{\mathrm{KEM}}_{1}}$ is the key space of ${\mathrm{KEM}}_{1}$.*

**Figure 7:**
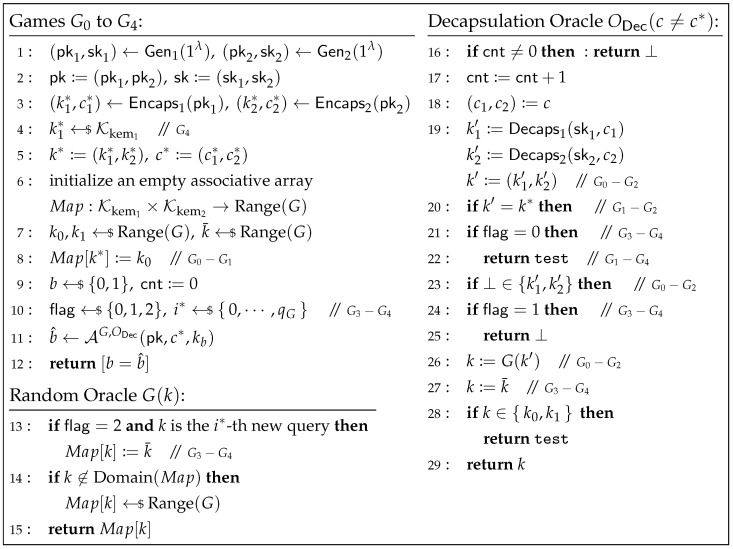
Games $G_{0}$ to $G_{4}$ for the proof of Theorem 3.

**Proof of Theorem 3.**  We define a sequence of games $G_{j}$ (for j=0,…,4) between the challenger and adversary A, as illustrated in Figure 7. Let $W_{j}$ be the event that A’s output $\hat{b}$ equals the challenger’s random bit *b* in game $G_{j}$.

**Game $G_{0}$.** This game is defined as in Figure 7. In this game, the challenger initializes an empty associative array $Map:K_{{\mathrm{kem}}_{1}}\times K_{{\mathrm{kem}}_{2}}\to \mathrm{Range}\left(G\right)$ to implement the random oracle *G*. Note that although the challenger initially selects a random $k_{0}\leftarrow_{\$}\mathrm{Range}\left(G\right)$, it immediately sets $Map\left[k^{*}\right]:=k_{0}$, which is equivalent to defining $G\left(k^{*}\right)=k_{0}$. Furthermore, although the challenger samples a random $\bar{k}\leftarrow_{\$}\mathrm{Range}\left(G\right)$, this value is not used in the subsequent game. Therefore, from the perspective of A, the behavior of the challenger in $G_{0}$ is consistent with that in the IND-1RCCA game for ${\mathrm{KEM}}_{int.}$. Thus, we have
  (1) $${\mathrm{Adv}}_{{\mathrm{KEM}}_{int.}}^{\text{IND-1RCCA}}\left(A\right)=\left|\mathrm{Pr}[W_{0}]-1/2\right|\;.$$
  **Game $G_{1}$.** In this game, the decapsulation oracle $O_{\mathrm{Dec}}$ explicitly handles the case where $k^{\prime }=k^{*}$ during decapsulation (as shown in lines 20 and 22 of Figure 7): when $k^{\prime }=k^{*}$, $O_{\mathrm{Dec}}$ directly returns test. From the perspective of A, this modification does not alter the return value of the decapsulation oracle. Hence,
  (2) $$\mathrm{Pr}\left[W_{1}\right]=\mathrm{Pr}\left[W_{0}\right]\;.$$
  **Game $G_{2}$.** In this game, the challenger no longer sets $Map\left[k^{*}\right]:=k_{0}$, i.e., line 8 in Figure 7 is removed. Let *Z* be the event that A queries the random oracle *G* on $k^{*}$. Note that if event *Z* does not occur, then $G(k^{*})$ is independent and random from the view of A, and thus $\mathrm{Pr}\left[W_{2}|\bar{Z}\right]=\mathrm{Pr}\left[W_{1}|\bar{Z}\right]$. By the Difference Lemma (Lemma 4), we have
  (3) $$\left|\mathrm{Pr}\left[W_{2}\right]-\mathrm{Pr}\left[W_{1}\right]\right|\leq \mathrm{Pr}\left[Z\right].$$
  Let $Z_{j}$ be the event that A queries the random oracle *G* on $\left(k_{1}^{*},\cdot \right)$ in game $G_{j}$. Clearly,
  (4) $$\mathrm{Pr}\left[Z\right]\leq \mathrm{Pr}\left[Z_{2}\right].$$
  Since both $k_{0}$ and $k_{1}$ are now independent and random from the view of A, we have
  (5) $$\mathrm{Pr}[W_{2}]=1/2.$$
  **Game $G_{3}$.** In this game, the decapsulation oracle $O_{\mathrm{Dec}}$ no longer relies on the specific value of $k^{\prime }$ to compute the return value, but instead determines the output of $O_{\mathrm{Dec}}$ through a “guessing” strategy. At the beginning of the game, the challenger randomly selects a $\mathrm{flag}\leftarrow_{\$}\left\{0,1,2\right\}$ to guess the possible scenarios for $k^{\prime }$ in the decapsulation oracle: flag=0 indicates that the challenger assumes $k^{\prime }=k^{*}$, in which case $O_{\mathrm{Dec}}$ directly returns test (as shown in lines 21 and 22 of Figure 7); flag=1 indicates that the challenger assumes $⊥\in \{k_{1}^{\prime },k_{2}^{\prime }\}$ in the decapsulation oracle simulation, in which case $O_{\mathrm{Dec}}$ directly returns ⊥ (as shown in lines 24 and 25 of Figure 7); and flag=2 corresponds to all other cases, where the challenger directly sets $k:=\bar{k}$ (as shown in line 27 of Figure 7), and then determines the return value of $O_{\mathrm{Dec}}$ based on *k*. Here, $\bar{k}\leftarrow_{\$}\mathrm{Range}\left(G\right)$ is a random value pre-selected by the challenger at the start of the game. In the case of flag=2, the challenger further modifies the random oracle *G* to ensure consistency between the simulation of the decapsulation oracle and the random oracle: if flag=2 and *k* is the $i^{*}$-th new query, then $Map\left[k\right]:=\bar{k}$ (see line 13 of Figure 7), where $i^{*}\leftarrow_{\$}\left\{0,\ldots ,q_{G}\right\}$ and $q_{G}$ is the bounded number of *G*-queries made by A.

Let *E* be the event that the challenger guesses correctly. We can see that:

- If $k^{\prime }=k^{*}$, the decapsulation oracle $O_{\mathrm{Dec}}$ in both games $G_{2}$ and $G_{3}$ returns test, hence $\mathrm{Pr}\left[Z_{2}\wedge k^{\prime }=k^{*}\right]=\mathrm{Pr}\left[Z_{3}\wedge k^{\prime }=k^{*}|E\right]$.
- If $⊥\in \{k_{1}^{\prime },k_{2}^{\prime }\}$, the decapsulation oracle $O_{\mathrm{Dec}}$ in both games $G_{2}$ and $G_{3}$ returns ⊥, hence $\mathrm{Pr}\left[Z_{2}\wedge ⊥\in \left\{k_{1}^{\prime },k_{2}^{\prime }\right\}\right]=\mathrm{Pr}\left[Z_{3}\wedge ⊥\in \left\{k_{1}^{\prime },k_{2}^{\prime }\right\}|E\right]$.
- If $k^{\prime }\neq k^{*}$ and $⊥\notin \{k_{1}^{\prime },k_{2}^{\prime }\}$, game $G_{2}$ sets $k:=G(k^{\prime })$, while game $G_{3}$ sets $k:=\bar{k}$. Note that in this case, the challenger in $G_{3}$ further randomly guesses that the adversary A might query $k^{\prime }$ to the random oracle *G* at the $i^{*}$-th new query, and sets $Map\left[k\right]:=\bar{k}$ ($i^{*}=0$ means A does not query $k^{\prime }$ to the random oracle *G*). If the challenger guesses correctly, then $O_{\mathrm{Dec}}$ in $G_{3}$ effectively returns $G(k^{\prime })$, making its behavior consistent with that of $O_{\mathrm{Dec}}$ in $G_{2}$. Since the probability that the challenger correctly guesses the index when A queries *G* on $k^{\prime }$ is $\mathrm{Pr}\left[\mathrm{guess}\;i^{*}\right]=1/\left(q_{G}+1\right)$, we have
  $$\begin{array}{cc} \mathrm{Pr}[Z_{2}\wedge k^{\prime }\neq k^{*}\wedge ⊥\notin \left\{k_{1}^{\prime },k_{2}^{\prime }\right\}] & =\mathrm{Pr}[Z_{3}\wedge k^{\prime }\neq k^{*}\wedge ⊥\notin \left\{k_{1}^{\prime },k_{2}^{\prime }\right\}|E\wedge \mathrm{guess}\;i^{*}]\\ \; & =\frac{\mathrm{Pr}[Z_{3}\wedge k^{\prime }\neq k^{*}\wedge ⊥\notin \left\{k_{1}^{\prime },k_{2}^{\prime }\right\}\wedge \mathrm{guess}\;i^{*}|E]}{\mathrm{Pr}[\mathrm{guess}\;i^{*}]}\\ \; & \leq \left(q_{G}+1\right)\mathrm{Pr}\left[Z_{3}\wedge k^{\prime }\neq k^{*}\wedge ⊥\notin \left\{k_{1}^{\prime },k_{2}^{\prime }\right\}|E\right]\;. \end{array}$$

Therefore,
  $$\begin{array}{cc} \mathrm{Pr}[Z_{3}|E] & \geq \mathrm{Pr}\left[Z_{2}\wedge k^{\prime }=k^{*}\right]+\mathrm{Pr}\left[Z_{2}\wedge ⊥\in \left\{k_{1}^{\prime },k_{2}^{\prime }\right\}\right]+\frac{\mathrm{Pr}[Z_{2}\wedge k^{\prime }\neq k^{*}\wedge ⊥\notin \left\{k_{1}^{\prime },k_{2}^{\prime }\right\}]}{q_{G}+1}\\ \; & \geq \mathrm{Pr}[Z_{2}]/q_{G}+1\;. \end{array}$$
  Since Pr[E]=1/3, we have
  (6) $$\mathrm{Pr}\left[Z_{3}\right]\geq \mathrm{Pr}\left[Z_{3}\wedge E\right]=\mathrm{Pr}\left[Z_{3}|E\right]\cdot \mathrm{Pr}\left[E\right]\geq \mathrm{Pr}[Z_{2}]/3(q_{G}+1)\;.$$
  **Game $G_{4}$.** In this game, the challenger samples $k_{1}^{*}\leftarrow_{\$}K_{{\mathrm{kem}}_{1}}$ uniformly at random (line 4 of Figure 7) to compute the challenge key $k^{*}$, instead of using $\left(k_{1}^{*},c_{1}^{*}\right)\leftarrow {\mathrm{Encaps}}_{1}\left({\mathrm{pk}}_{1}\right)$. We now show that if A can distinguish between $G_{3}$ and $G_{4}$, we can construct an adversary B against the IND-CPA security of ${\mathrm{KEM}}_{1}$.

Specifically, upon receiving $\left({\mathrm{pk}}_{1},k_{b^{\prime }},c_{1}^{*}\right)$ from the IND-CPA challenger of ${\mathrm{KEM}}_{1}$, where $b^{\prime }$ is a random bit chosen by the challenger, B sets $k_{1}^{*}:=k_{b^{\prime }}$, computes $\left({\mathrm{pk}}_{2},{\mathrm{sk}}_{2}\right)\leftarrow {\mathrm{Gen}}_{2}\left(1^{\lambda }\right)$ and $\left(k_{2}^{*},c_{2}^{*}\right)\leftarrow {\mathrm{Encaps}}_{2}\left({\mathrm{pk}}_{2}\right)$, and sets $\mathrm{pk}:=({\mathrm{pk}}_{1},{\mathrm{pk}}_{2})$, $k^{*}:=\left(k_{1}^{*},k_{2}^{*}\right)$, and $c^{*}:=\left(c_{1}^{*},c_{2}^{*}\right)$. It then samples $k\leftarrow_{\$}\mathrm{Range}\left(G\right)$ and sends $\left(\mathrm{pk},c^{*},k\right)$ to A. The random oracle *G* and the decapsulation oracle $O_{\mathrm{Dec}}$ are simulated by B consistently with their behavior in $G_{3}$. At the end of the game, if $k_{b^{\prime }}\in \mathrm{Domain}\left(Map\right)$, B outputs ${\hat{b}}^{\prime }=1$; otherwise, it outputs ${\hat{b}}^{\prime }=0$.

Note that when $b^{\prime }=0$, $k_{b^{\prime }}$ is the real key, and B’s behavior is identical to that of the challenger in $G_{3}$, so $\mathrm{Pr}\left[{\hat{b}}^{\prime }=1|b^{\prime }=0\right]=\mathrm{Pr}\left[Z_{3}\right]$; when $b^{\prime }=1$, $k_{b^{\prime }}$ is a random key, and B’s behavior is identical to that of the challenger in $G_{4}$, so $\mathrm{Pr}\left[{\hat{b}}^{\prime }=1|b^{\prime }=1\right]=\mathrm{Pr}\left[Z_{4}\right]$. Therefore, we have

(7) $$\begin{array}{cc} {\mathrm{Adv}}_{{\mathrm{KEM}}_{1}}^{\text{IND-CPA}}\left(B\right) & \geq \;|\mathrm{Pr}[b^{\prime }={\hat{b}}^{\prime }]-1/2|\\ \; & =\;|\mathrm{Pr}\left[{\hat{b}}^{\prime }=0\wedge b^{\prime }=0\right]+\mathrm{Pr}\left[{\hat{b}}^{\prime }=1\wedge b^{\prime }=1\right]-1/2|\\ \; & =\;|\mathrm{Pr}\left[{\hat{b}}^{\prime }=0|b^{\prime }=0\right]/2+\mathrm{Pr}\left[{\hat{b}}^{\prime }=1|b^{\prime }=1\right]/2-1/2|\\ \; & =\;|\left(1-\mathrm{Pr}\left[{\hat{b}}^{\prime }=1|b^{\prime }=0\right]\right)/2+\mathrm{Pr}\left[{\hat{b}}^{\prime }=1|b^{\prime }=1\right]/2-1/2|\\ \; & =\;|\mathrm{Pr}\left[Z_{4}\right]-\mathrm{Pr}\left[Z_{3}\right]|/2\;. \end{array}$$

Since in $G_{4}$, $k_{1}^{*}$ is independent and random from the view of A, we have

(8) $$\mathrm{Pr}\left[Z_{4}\right]=q_{G}/|K_{{\mathrm{kem}}_{1}}|\;.$$

Combining Equations (1)–(8), we obtain

$${\mathrm{Adv}}_{{\mathrm{KEM}}_{int.}}^{\text{IND-1RCCA}}\left(A\right)\leq 6\left(q_{G}+1\right)\cdot {\mathrm{Adv}}_{{\mathrm{KEM}}_{1}}^{\text{IND-CPA}}\left(B\right)+3q_{G}\left(q_{G}+1\right)/|K_{{\mathrm{kem}}_{1}}|\;.$$

   □

#### 3.2.2. Security of the *Intermediate* KEM in the QROM

**Theorem** **4** (IND-1RCCA security of the hybrid ${\mathrm{KEM}}_{int.}$ in the QROM)**.** *Let ${\mathrm{KEM}}_{int.}:=T_{int.}\left[\mathrm{Comb}\left[{\mathrm{KEM}}_{1},{\mathrm{KEM}}_{2}\right],G\right]$ (Comb and $T_{int.}$ are defined in Figure 2 and Figure 4) with G modeled as a quantum-accessible random oracle. If ${\mathrm{KEM}}_{1}$ is IND-CPA secure, then ${\mathrm{KEM}}_{int.}$ is IND-1RCCA secure. Specifically, any efficient adversary A against ${\mathrm{KEM}}_{int.}$ implies an efficient adversary B against ${\mathrm{KEM}}_{1}$ with*

$${\mathrm{Adv}}_{{\mathrm{KEM}}_{int.}}^{\text{IND-1RCCA}}\left(A\right)\leq 10\left(q_{G}+1\right)\sqrt{2{\mathrm{Adv}}_{{\mathrm{KEM}}_{1}}^{\text{IND-CPA}}\left(B\right)+{\left(q_{G}+1\right)}^{2}/|K_{{\mathrm{kem}}_{1}}|+1/\left|G\right|}\;,$$

*where $q_{G}$ bounds A’s queries to G, $K_{{\mathrm{KEM}}_{1}}$ is the key space of ${\mathrm{KEM}}_{1}$, and |G| is the output space size of G.*

**Figure 8:**
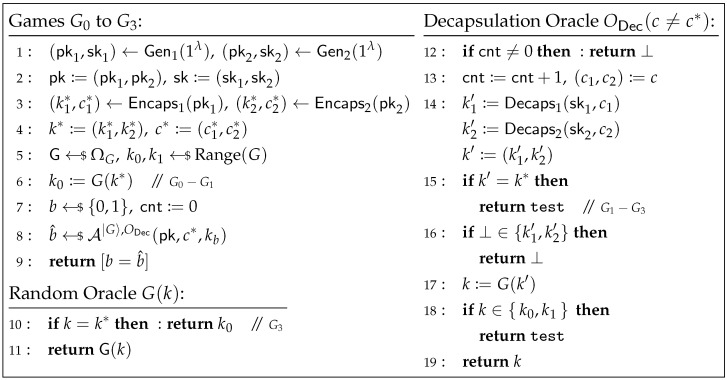
Games $G_{0}$ to $G_{3}$ for the proof of Theorem 4.

**Proof of Theorem 4.**  We define a sequence of games $G_{j}$ (for j=0,…,3) between the challenger and adversary A, as depicted in Figure 8. Let $W_{j}$ denote the event that A’s output $\hat{b}$ equals the challenger’s random bit *b* in game $G_{j}$.

**Game $G_{0}$.** This game is defined as in Figure 8. In this game, the challenger implements the random oracle *G* by sampling a random function $G\leftarrow_{\$}\Omega_{G}$. Note that although the challenger initially selects a random $k_{0}\leftarrow_{\$}\mathrm{Range}\left(G\right)$ (as shown in line 5 of Figure 8), it is immediately overwritten by setting $k_{0}:=G\left(k^{*}\right)$ (i.e., line 6). Therefore, from the perspective of A, the behavior of the challenger in $G_{0}$ is identical to that in the IND-1RCCA game for ${\mathrm{KEM}}_{int.}$. Hence, we have
  (9) $${\mathrm{Adv}}_{{\mathrm{KEM}}_{int.}}^{\text{IND-1RCCA}}\left(A\right)=\left|\mathrm{Pr}[W_{0}]-1/2\right|\;.$$
  **Game $G_{1}$.** In this game, the decapsulation oracle $O_{\mathrm{Dec}}$ explicitly handles the case where $k^{\prime }=k^{*}$ (see line 15 of Figure 8): if $k^{\prime }=k^{*}$, it directly returns test. Note that this operation does not alter the behavior of $O_{\mathrm{Dec}}$. Therefore,
  (10) $$\mathrm{Pr}\left[W_{1}\right]=\mathrm{Pr}\left[W_{0}\right]\;.$$
  **Game $G_{2}$.** In this game, the challenger no longer resets $k_{0}:=G\left(k^{*}\right)$, i.e., line 6 in Figure 8 is removed. Since both $k_{0}$ and $k_{1}$ are now independent from the view of A, we have
  (11) $$\mathrm{Pr}[W_{2}]=1/2\;.$$
  **Game $G_{3}$.** In this game, the challenger adds an additional operation to the simulation of the random oracle *G*: if the query $k=k^{*}$, it returns $k_{0}$. Note that this is equivalent to setting $G\left(k^{*}\right):=k_{0}$. From the perspective of A, the behavior of the challenger is now consistent with that in $G_{1}$. Hence,
  (12) $$\mathrm{Pr}\left[W_{3}\right]=\mathrm{Pr}\left[W_{1}\right]\;.$$

Observe that compared to $G_{2}$, the challenger can be viewed as implementing the random oracle *G* using a new random function $G^{\prime }\in \Omega_{G}$, where $G^{\prime }\left(k\right)=G\left(k\right)$ for all $k\neq k^{*}$ and $G^{\prime }\left(k^{*}\right)=k_{0}$. To bound $|\mathrm{Pr}\left[W_{3}\right]-\mathrm{Pr}\left[W_{2}\right]|$ using the DS-O2H Lemma (Lemma 1), we first construct two oracle algorithms $A^{|G\rangle ,O_{\mathrm{Dec}}}\left(z\right)$ and $A^{|G^{\prime }\rangle ,O_{\mathrm{Dec}}}\left(z\right)$ that simulate games $G_{2}$ and $G_{3}$, respectively. Here, $z:=(\mathrm{pk},c^{*},k_{0},k_{1},b)$, and $\left(\mathrm{pk},c^{*},k_{0},k_{1},b\right)$ is generated consistently with game $G_{2}$: first compute $\left({\mathrm{pk}}_{1},{\mathrm{sk}}_{1}\right)\leftarrow {\mathrm{Gen}}_{1}\left(1^{\lambda }\right)$ and $\left({\mathrm{pk}}_{2},{\mathrm{sk}}_{2}\right)\leftarrow {\mathrm{Gen}}_{2}\left(1^{\lambda }\right)$, then set $\mathrm{pk}:=({\mathrm{pk}}_{1},{\mathrm{pk}}_{2})$; next compute $\left(k_{1}^{*},c_{1}^{*}\right)\leftarrow {\mathrm{Encaps}}_{1}\left({\mathrm{pk}}_{1}\right)$ and $\left(k_{2}^{*},c_{2}^{*}\right)\leftarrow {\mathrm{Encaps}}_{2}\left({\mathrm{pk}}_{2}\right)$, and set $c^{*}:=\left(c_{1}^{*},c_{2}^{*}\right)$; finally, sample $k_{0},k_{1}\leftarrow_{\$}\mathrm{Range}\left(G\right)$ and $b\leftarrow_{\$}\left\{0,1\right\}$ uniformly at random. Let $G\leftarrow_{\$}\Omega_{G}$, and define $G^{\prime }\in \Omega_{G}$ such that $G^{\prime }\left(k\right)=G\left(k\right)$ for all $k\neq k^{*}$ and $G^{\prime }\left(k^{*}\right)=k_{0}$, where $k^{*}:=\left(k_{1}^{*},k_{2}^{*}\right)$. The algorithm $A^{|G\rangle ,O_{\mathrm{Dec}}}\left(z\right)$ (resp. $A^{|G^{\prime }\rangle ,O_{\mathrm{Dec}}}\left(z\right)$) simulates the random oracle *G* as described in game $G_{2}$ (resp. $G_{3}$), runs $\hat{b}\leftarrow A^{|G\rangle ,O_{\mathrm{Dec}}}\left(\mathrm{pk},c^{*},k_{b}\right)$, and outputs $\left[b=\hat{b}\right]$. It is not hard to see that

(13) $$\mathrm{Pr}\left[1\leftarrow A^{|G\rangle ,O_{\mathrm{Dec}}}\left(z\right)\right]=\mathrm{Pr}\left[W_{2}\right]\;\mathrm{and}\;\mathrm{Pr}\left[1\leftarrow A^{|G^{\prime }\rangle ,O_{\mathrm{Dec}}}\left(z\right)\right]=\mathrm{Pr}\left[W_{3}\right]\;.$$

Since G and $G^{\prime }$ differ only at $k^{*}$, by the DS-O2H Lemma (Lemma 1), there exists an oracle algorithm $B^{|G\rangle ,|G^{\prime }\rangle ,O_{\mathrm{Dec}}}\left(z\right)$ such that

(14) $$|\mathrm{Pr}\left[1\leftarrow A^{|G\rangle ,O_{\mathrm{Dec}}}\left(z\right)\right]-\mathrm{Pr}\left[1\leftarrow A^{|G^{\prime }\rangle ,O_{\mathrm{Dec}}}\left(z\right)\right]|\;\leq 2\sqrt{\mathrm{Pr}[k^{*}=\hat{k}:\hat{k}\leftarrow B^{|G\rangle ,|G^{\prime }\rangle ,O_{\mathrm{Dec}}}\left(z\right)]}\;,$$

where *B* makes at most $q_{G}$ queries to |G〉 and $q_{G}$ is the upper bound on the number of *G*-queries made by A. Note that in the simulation of $O_{\mathrm{Dec}}$ in $G_{2}$ and $G_{3}$, the query $k^{\prime }$ to the random oracle *G* (i.e., line 17 of Figure 8) is not equal to $k^{*}$ (since the case $k^{\prime }=k^{*}$ has been handled at line 15 by directly returning test). Therefore, the simulation of $O_{\mathrm{Dec}}$ is consistent in $G_{2}$ and $G_{3}$, and is independent of whether *G* is simulated using G or $G^{\prime }$. Thus, when applying the DS-O2H Lemma, $O_{\mathrm{Dec}}$ can be treated as an internal oracle of *A*.

**Figure 9:**
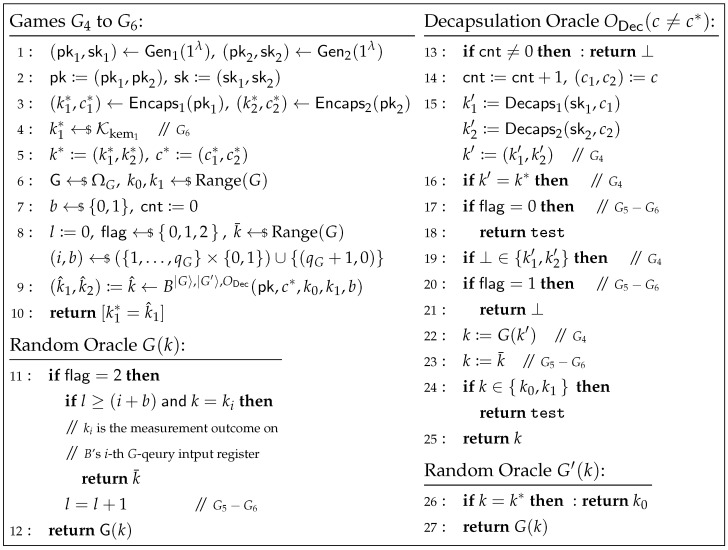
Games $G_{4}$ to $G_{6}$ for the proof of Theorem 4.

Next, we define a sequence of games $G_{j}$ (for j=4,…,6) played between a challenger and the oracle algorithm *B*, as shown in Figure 9. Here, $k_{1}^{*}$ is the key computed by the challenger for ${\mathrm{KEM}}_{1}$, and $\hat{k}=\left({\hat{k}}_{1},{\hat{k}}_{2}\right)$ is the key output by *B*. Let $Z_{j}$ denote the event that $k_{1}^{*}={\hat{k}}_{1}$ in game $G_{j}$.

**Game $G_{4}$.** In this game, the challenger samples a random function $G\leftarrow_{\$}\Omega_{G}$ to simulate the random oracles *G* and $G^{\prime }$: G(k) directly returns G(k); $G^{\prime }\left(k^{*}\right)$ returns $k_{0}$, while $G^{\prime }\left(k\neq k^{*}\right)$ returns G(k) (i.e., G(k)). This is equivalent to *B* having direct access to G and $G^{\prime }$. Note that although the challenger samples additional values in line 8, they are never actually used in the game. Therefore, we have
  (15) $$\mathrm{Pr}\left[k^{*}=\hat{k}:\hat{k}\leftarrow B^{|G\rangle ,|G^{\prime }\rangle ,O_{\mathrm{Dec}}}\left(z\right)\right]\leq \mathrm{Pr}\left[k_{1}^{*}={\hat{k}}_{1}:\left({\hat{k}}_{1},{\hat{k}}_{2}\right):=\hat{k}\leftarrow B^{|G\rangle ,|G^{\prime }\rangle ,O_{\mathrm{Dec}}}\left(z\right)\right]=\mathrm{Pr}\left[Z_{4}\right]\;.$$
**Game $G_{5}$.** In this game, the challenger employs a “guessing” strategy to simulate the decapsulation oracle $O_{\mathrm{Dec}}$, thereby eliminating its dependency on sk. The challenger first selects a random $\mathrm{flag}\leftarrow_{\$}\left\{0,1,2\right\}$, and for the $k^{\prime }=\left(k_{1}^{\prime },k_{2}^{\prime }\right)$ in $O_{\mathrm{Dec}}$: when flag=0, the challenger guesses $k^{\prime }=k^{*}$ and directly outputs test; when flag=1, the challenger guesses $⊥\in \{k_{1}^{\prime },k_{2}^{\prime }\}$ and directly outputs ⊥; when flag=2, the challenger guesses that $k^{\prime }$ falls into other cases, i.e., $k^{\prime }\neq k^{*}$ and $⊥\notin \{k_{1}^{\prime },k_{2}^{\prime }\}$. In the last case, the challenger sets $k:=\bar{k}$, where $\bar{k}\leftarrow_{\$}\mathrm{Range}\left(G\right)$ is a random value pre-sampled by the challenger. If $k\in \{k_{0},k_{1}\}$, $O_{\mathrm{Dec}}$ returns ⊥; otherwise, it returns *k*.

In the case of flag=2, the challenger further modifies the random oracle *G* as follows: for the *l*-th *G*-query where l≥(i+b), *G* uses $\bar{k}$ as the return value for the query $k=k_{i}$. Here, $\left(i,b\right)\leftarrow_{\$}\left(\left\{1,\ldots ,q_{G}\right\}\times \left\{0,1\right\}\right)\cup \left\{\left(q_{G}+1,0\right)\right\}$ is a random value pre-sampled by the challenger, and $k_{i}$ is the measurement outcome of the challenger’s input register for *B*’s *i*-th *G*-query.

Let *E* be the event that the challenger guesses the correct flag. We can observe that:

- If $k^{\prime }=k^{*}$, the decapsulation oracle $O_{\mathrm{Dec}}$ in both games $G_{4}$ and $G_{5}$ returns test, hence $\mathrm{Pr}\left[Z_{4}\wedge k^{\prime }=k^{*}\right]=\mathrm{Pr}\left[Z_{5}\wedge k^{\prime }=k^{*}|E\right]$.
- If $⊥\in \{k_{1}^{\prime },k_{2}^{\prime }\}$, the decapsulation oracle $O_{\mathrm{Dec}}$ in both games $G_{4}$ and $G_{5}$ returns ⊥, hence $\mathrm{Pr}\left[Z_{4}\wedge ⊥\in \left\{k_{1}^{\prime },k_{2}^{\prime }\right\}\right]=\mathrm{Pr}\left[Z_{5}\wedge ⊥\in \left\{k_{1}^{\prime },k_{2}^{\prime }\right\}|E\right]$.
- If $k^{\prime }\neq k^{*}$ and $⊥\notin \{k_{1}^{\prime },k_{2}^{\prime }\}$, we can use Lemma 3 to derive that $\mathrm{Pr}\left[Z_{4}\wedge k^{\prime }\neq k^{*}\wedge ⊥\notin \left\{k_{1}^{\prime },k_{2}^{\prime }\right\}\right]\leq 8{\left(q_{G}+1\right)}^{2}(\mathrm{Pr}\left[Z_{5}\wedge k^{\prime }\neq k^{*}\wedge ⊥\notin \left\{k_{1}^{\prime },k_{2}^{\prime }\right\}|E\right]+1/\left|G|\right)$ as follows, where |G| is the output space size of *G*.

Conditioned on $k^{\prime }\neq k^{*}$ and $⊥\notin \{k_{1}^{\prime },k_{2}^{\prime }\}$, we can construct an oracle algorithm $C^{|G\rangle }$ that generates $\left(\mathrm{pk},\mathrm{sk},k^{*},c^{*},k_{0},k_{1},b\right)$ and runs $B^{|G\rangle ,|G^{\prime }\rangle ,O_{\mathrm{Dec}}}$ as the challenger does in $G_{4}$. Let $\bar{c}=\left({\bar{c}}_{1},{\bar{c}}_{2}\right)$ be the decapsulation oracle query made by *B*, and define $k_{1}^{\prime }:={\mathrm{Decaps}}_{1}\left({\mathrm{sk}}_{1},{\bar{c}}_{1}\right)$, $k_{2}^{\prime }:={\mathrm{Decaps}}_{2}\left({\mathrm{sk}}_{2},{\bar{c}}_{2}\right)$, $k^{\prime }:=\left(k_{1}^{\prime },k_{2}^{\prime }\right)$. Define $x:=k^{\prime }$, y:=G(x), and $z:=\left(z_{1},z_{2},z_{3}\right)=\left(k,k_{1}^{*},{\hat{k}}_{1}\right)$, where *k* is the value used in $O_{\mathrm{Dec}}$ to determine its final output (i.e., the *k* in line 24 of Figure 9). If *k* is undefined, i.e., when $k^{\prime }=k^{*}$ or $⊥\in \{k_{1}^{\prime },k_{2}^{\prime }\}$, we set k:=⊥. Define the predicates $V_{1}\left(x,y,z\right):=\left[y=z_{1}\right]$ and $V_{2}:=\left[z_{2}=z_{3}\right]$. Let $V:=V_{1}\wedge V_{2}$. Note that in game $G_{4}$, when $k^{\prime }\neq k^{*}$ and $⊥\notin \{k_{1}^{\prime },k_{2}^{\prime }\}$, $V_{1}\left(x,y,z\right)$ always holds. Therefore, we can see that

$$\begin{array}{cc} \; & \mathrm{Pr}\left[Z_{4}|k^{\prime }\neq k^{*}\wedge ⊥\notin \left\{k_{1}^{\prime },k_{2}^{\prime }\right\}\right]=\underset{G}{\mathrm{Pr}}\left[V\left(x,y,z\right)=1:\left(x,z\right)\leftarrow C^{|G\rangle }\right]\\ \; & \;\;\;\;\;\;=\sum_{x_{0}\in K_{{\mathrm{kem}}_{1}}\times K_{{\mathrm{kem}}_{2}}}\underset{G}{\mathrm{Pr}}\mathrm{Pr}\left[x=x_{0}\wedge V\left(x,y,z\right)=1:\left(x,z\right)\leftarrow C^{|G\rangle }\right]\;. \end{array}$$

It is important to note that in game $G_{4}$, besides the $q_{G}$ oracle *G* queries made by the oracle algorithm *B*, the decapsulation oracle $O_{\mathrm{Dec}}$ implicitly makes at most one additional (classical) query to $G(k^{\prime })$ (i.e., line 22 in Figure 9). Thus, *C* makes a total of $q_{G}+1$ random oracle *G* queries. Unless otherwise specified, the *G* queries mentioned below exclude the implicit *G* query in $O_{\mathrm{Dec}}$. Let $S^{C}\left(\Theta \right)$ be the oracle algorithm that always uses Θ to respond to *C*’s implicit *G* query $G(k^{\prime })$. This algorithm randomly samples $\left(i,b\right)\leftarrow_{\$}\left(\left\{1,\ldots ,q_{G}\right\}\times \left\{0,1\right\}\right)\cup \left\{\left(q_{G}+1,0\right)\right\}$, runs $C^{|G\rangle }$ until just before *C*’s *i*-th *G* query (excluding the implicit query), measures *C*’s input register to obtain *x*, continues running $C^{|G\rangle }$ until its (i+b)-th *G* query, reprograms *G* to $G_{x\Theta }$ (where $G_{x\Theta }\left(x^{\prime }\right)=G\left(x^{\prime }\right)$ for all $x^{\prime }\neq x$ and $G_{x\Theta }\left(x\right)=\Theta$), and continues running $C^{|G_{x\Theta }\rangle }$ until it outputs *z*. Let y:=Θ and $z:=\left(z_{1},z_{2},z_{3}\right)=\left(k,k_{1}^{*},{\hat{k}}_{1}\right)$, where *k* is the value used in $O_{\mathrm{Dec}}$ to determine its final output (i.e., the *k* in line 24 of Figure 9). Note that $V_{1}\left(x,y,z\right)=\left[y=z_{1}\right]$ still holds here. Therefore, when $\Theta \leftarrow_{\$}\mathrm{Range}\left(G\right)$, under the condition $k^{\prime }\neq k^{*}$ and $⊥\notin \{k_{1}^{\prime },k_{2}^{\prime }\}$, $S^{C}\left(\Theta \right)$ perfectly simulates game $G_{5}$ when flag=2. Hence,

$$\begin{array}{cc} \; & \mathrm{Pr}\left[Z_{5}|k^{\prime }\neq k^{*}\wedge ⊥\notin \left\{k_{1}^{\prime },k_{2}^{\prime }\right\}\wedge E\right]=\underset{G,\Theta }{\mathrm{Pr}}\left[V\left(x,\Theta ,z\right)=1:\left(x,z\right)\leftarrow S^{C}\left(\Theta \right)\right]\\ \; & \;\;\;\;\;\;=\sum_{x_{0}\in K_{{\mathrm{kem}}_{1}}\times K_{{\mathrm{kem}}_{2}}}\underset{G,\Theta }{\mathrm{Pr}}\left[x=x_{0}\wedge V\left(x,\Theta ,z\right)=1:\left(x,z\right)\leftarrow S^{C}\left(\Theta \right)\right]\;. \end{array}$$

By Lemma 3, we have

$$\begin{array}{cc} \; & \;\sum_{x_{0}\in K_{{\mathrm{kem}}_{1}}\times K_{{\mathrm{kem}}_{2}}}\underset{G}{\mathrm{Pr}}\mathrm{Pr}\left[x=x_{0}\wedge V\left(x,y,z\right)=1:\left(x,z\right)\leftarrow C^{|G\rangle }\right]\\ \; & \leq 2{\left(2q_{G}+1\right)}^{2}\sum_{x_{0}\in K_{{\mathrm{kem}}_{1}}\times K_{{\mathrm{kem}}_{2}}}\underset{G,\Theta }{\mathrm{Pr}}\left[x=x_{0}\wedge V\left(x,\Theta ,z\right)=1:\left(x,z\right)\leftarrow S^{C}\left(\Theta \right)\right]+\frac{8{\left(q_{G}+1\right)}^{2}}{\left|G\right|}, \end{array}$$

where |G| is the size of the output space of *G*. Therefore, we have

$$\begin{array}{cc} \; & \mathrm{Pr}\left[Z_{4}\wedge k^{\prime }\neq k^{*}\wedge ⊥\notin \left\{k_{1}^{\prime },k_{2}^{\prime }\right\}\right]=\mathrm{Pr}\left[Z_{4}|k^{\prime }\neq k^{*}\wedge ⊥\notin \left\{k_{1}^{\prime },k_{2}^{\prime }\right\}\right]\mathrm{Pr}\left[k^{\prime }\neq k^{*}\wedge ⊥\notin \left\{k_{1}^{\prime },k_{2}^{\prime }\right\}\right]\\ \; & \leq \left(8{\left(q_{G}+1\right)}^{2}\left(\mathrm{Pr}\left[Z_{5}|k^{\prime }\neq k^{*}\wedge ⊥\notin \left\{k_{1}^{\prime },k_{2}^{\prime }\right\}\wedge E\right]+\frac{1}{\left|G\right|}\right)\right)\mathrm{Pr}\left[k^{\prime }\neq k^{*}\wedge ⊥\notin \left\{k_{1}^{\prime },k_{2}^{\prime }\right\}\right]\\ \; & \leq 8{\left(q_{G}+1\right)}^{2}\left(\mathrm{Pr}[Z_{5}\wedge k^{\prime }\neq k^{*}\wedge ⊥\notin \left\{k_{1}^{\prime },k_{2}^{\prime }\right\}|E]+1/\left|G\right|\right). \end{array}$$

Consequently, we have

(16) $$\begin{array}{cc} \mathrm{Pr}[W_{5}] & \geq (\mathrm{Pr}\left[Z_{5}\wedge k^{\prime }=k^{*}|E\right]+\mathrm{Pr}\left[Z_{5}\wedge ⊥\in \left\{k_{1}^{\prime },k_{2}^{\prime }\right\}|E\right]\\ \; & \;\;\;\;\;\;\;\;+\mathrm{Pr}\left[Z_{5}\wedge k^{\prime }\neq k^{*}\wedge ⊥\notin \left\{k_{1}^{\prime },k_{2}^{\prime }\right\}|E\right])\mathrm{Pr}\left[E\right]\\ \; & \geq \left(\mathrm{Pr}\left[Z_{4}\wedge k^{\prime }=k^{*}\right]+\mathrm{Pr}\left[Z_{4}\wedge ⊥\in \left\{k_{1}^{\prime },k_{2}^{\prime }\right\}\right]\right)\mathrm{Pr}\left[E\right]\\ \; & \;\;\;\;\;\;\;\;+\frac{\mathrm{Pr}\left[Z_{4}\wedge k^{\prime }\neq k^{*}\wedge ⊥\notin \left\{k_{1}^{\prime },k_{2}^{\prime }\right\}\right]\mathrm{Pr}\left[E\right]}{8{\left(q_{G}+1\right)}^{2}}-\frac{1}{\left|G\right|}\\ \; & \geq \mathrm{Pr}[Z_{4}]/24{\left(q_{G}+1\right)}^{2}-1/\left|G\right|\;. \end{array}$$

**Game $G_{6}$.** In this game, the challenger replaces $k_{1}^{*}$ in $k^{*}:=\left(k_{1}^{*},k_{2}^{*}\right)$ with a random $k_{1}^{*}\leftarrow_{\$}K_{{\mathrm{kem}}_{1}}$ (as shown in line 4 of Figure 9). Note that in both games $G_{5}$ and $G_{6}$, the simulation of the decapsulation oracle $O_{\mathrm{Dec}}$ no longer uses sk. We proceed to construct a new adversary B against the IND-CPA security of ${\mathrm{KEM}}_{1}$.

Specifically, upon receiving $\left({\mathrm{pk}}_{1},k_{b^{\prime }},c_{1}^{*}\right)$ from the IND-CPA challenger of ${\mathrm{KEM}}_{1}$, where $b^{\prime }$ is a random bit chosen by the IND-CPA challenger of ${\mathrm{KEM}}_{1}$, B sets $k_{1}^{*}:=k_{b^{\prime }}$, then computes $\left({\mathrm{pk}}_{2},{\mathrm{sk}}_{2}\right)\leftarrow {\mathrm{Gen}}_{2}\left(1^{\lambda }\right)$ and $\left(k_{2}^{*},c_{2}^{*}\right)\leftarrow {\mathrm{Encaps}}_{2}\left({\mathrm{pk}}_{2}\right)$. It sets $\mathrm{pk}:=({\mathrm{pk}}_{1},{\mathrm{pk}}_{2})$, $k^{*}:=\left(k_{1}^{*},k_{2}^{*}\right)$, and $c^{*}:=\left(c_{1}^{*},c_{2}^{*}\right)$, samples $k_{0},k_{1}\leftarrow_{\$}\mathrm{Range}\left(G\right)$ and $b\leftarrow_{\$}\left\{0,1\right\}$ uniformly at random, and defines $z:=(\mathrm{pk},c^{*},k_{0},k_{1},b)$. It then runs $B^{|G\rangle ,|G^{\prime }\rangle ,O_{\mathrm{Dec}}}\left(z\right)$, where the random oracles *G*, $G^{\prime }$ and the decapsulation oracle $O_{\mathrm{Dec}}$ are simulated consistently with the challenger’s behavior in $G_{5}$. When $B^{|G\rangle ,|G^{\prime }\rangle ,O_{\mathrm{Dec}}}\left(z\right)$ outputs $\hat{k}=\left({\hat{k}}_{1},{\hat{k}}_{2}\right)$, B outputs ${\hat{b}}^{\prime }:=\left[k_{1}^{*}={\hat{k}}_{1}\right]$.

Note that when $b^{\prime }=0$, $k_{b^{\prime }}$ is the real key, and thus B’s behavior is identical to that of the challenger in $G_{5}$, so $\mathrm{Pr}\left[{\hat{b}}^{\prime }=1|b^{\prime }=0\right]=\mathrm{Pr}\left[Z_{5}\right]$; when $b^{\prime }=1$, $k_{b^{\prime }}$ is a random key, and thus B’s behavior is identical to that of the challenger in $G_{6}$, so $\mathrm{Pr}\left[{\hat{b}}^{\prime }=1|b^{\prime }=1\right]=\mathrm{Pr}\left[Z_{6}\right]$. Hence, we have

(17) $$\begin{array}{cc} {\mathrm{Adv}}_{{\mathrm{KEM}}_{1}}^{\text{IND-CPA}}\left(B\right) & \geq \;|\mathrm{Pr}[b^{\prime }={\hat{b}}^{\prime }]-1/2|\\ \; & =\;|\mathrm{Pr}\left[{\hat{b}}^{\prime }=0\wedge b^{\prime }=0\right]+\mathrm{Pr}\left[{\hat{b}}^{\prime }=1\wedge b^{\prime }=1\right]-1/2|\\ \; & =\;|\mathrm{Pr}\left[{\hat{b}}^{\prime }=0|b^{\prime }=0\right]/2+\mathrm{Pr}\left[{\hat{b}}^{\prime }=1|b^{\prime }=1\right]/2-1/2|\\ \; & =\;|\left(1-\mathrm{Pr}\left[{\hat{b}}^{\prime }=1|b^{\prime }=0\right]\right)/2+\mathrm{Pr}\left[{\hat{b}}^{\prime }=1|b^{\prime }=1\right]/2-1/2|\\ \; & =\;|\mathrm{Pr}\left[Z_{6}\right]-\mathrm{Pr}\left[Z_{5}\right]|/2\;. \end{array}$$

Since in game $G_{6}$, $k_{1}^{*}$ is independent of the random oracle *G*, the decapsulation oracle $O_{\mathrm{Dec}}$, and the input *z* to *B*, and *B* makes at most $q_{G}+1$ random oracle *G* queries, by Lemma 2, we have

(18) $$\mathrm{Pr}\left[Z_{6}\right]\leq {\left(q_{G}+1\right)}^{2}/|K_{{\mathrm{kem}}_{1}}|\;.$$

Combining Equations (9)–(18), we obtain

$${\mathrm{Adv}}_{{\mathrm{KEM}}_{int.}}^{\text{IND-1RCCA}}\left(A\right)\leq 10\left(q_{G}+1\right)\sqrt{2{\mathrm{Adv}}_{{\mathrm{KEM}}_{1}}^{\text{IND-CPA}}\left(B\right)+{\left(q_{G}+1\right)}^{2}/|K_{{\mathrm{kem}}_{1}}|+1/\left|G\right|}\;.$$

   □

**Remarks on Theorem 4.**  We should note that since random oracles are usually implemented using public hash functions in practice, a quantum adversary can query them in superposition “off-line”, which is captured by the QROM. Therefore, a security reduction in the QROM, which bounds a quantum adversary’s advantage against the obtained scheme by the advantage against a underlying primitive, ensures that the scheme’s security holds against quantum adversaries, assuming the primitive is secure. This is precisely the approach taken in the proof of Theorem 4, where the adversary is given quantum access to oracle *G*, and the analysis from Game $G_{3}$ to $G_{5}$ addresses the implications of this access. Theorem 4 concludes that even in the presence of such a quantum adversary A, ${\mathrm{KEM}}_{int.}$ is secure (i.e., ${\mathrm{Adv}}_{{\mathrm{KEM}}_{int.}}^{\text{IND-1RCCA}}\left(A\right)$ is negligible), as long as the underlying ${\mathrm{KEM}}_{1}$ is secure (i.e., ${\mathrm{Adv}}_{{\mathrm{KEM}}_{1}}^{\text{IND-CPA}}\left(B\right)$ is negligible), and provided the key space size $|K_{{\mathrm{kem}}_{1}}|$ and the output space size |G| of the hash function *G* are sufficiently large.

#### 3.2.3. Security of TLS 1.3 Hybrid Handshake from IND-CPA Secure Base KEM

Combining Theorems 3 and 4 with the results from [12], we finally establish the complete security of TLS 1.3 handshake with hybrid key exchange from IND-CPA secure KEMs.

**Theorem** **5** (Security of TLS 1.3 handshake with hybrid key exchange from IND-CPA secure KEM)**.** *If the other components in TLS 1.3 handshake are secure (as defined in [12]), then the entire TLS 1.3 with hybrid key exchange is secure, provided that at least one base KEM is IND-CPA secure.*

**Proof of Theorem 5.**  This result follows by combining Theorem 3 with Theorem 4.1 of [12] (which establishes that IND-1RCCA security of the *intermediate* KEM implies the special IND-1CCA-1MAC security in the ROM), and Theorem 4 with Theorem 4.2 of the same reference (which proves the same implication in the QROM), and finally applying Theorems 6.2 and 6.3 of [12] (which show that IND-1CCA-1MAC security of the *intermediate* KEM implies multi-stage security for the TLS 1.3 handshake).    □

## 4. Experimental Evaluation and Analysis

We have shown that CPA-secure KEMs suffice for the TLS 1.3 hybrid handshake. In contrast, current deployments (including IETF drafts [21,22]) rely on stronger IND-CCA secure KEMs like ML-KEM, which use the Fujisaki-Okamoto (FO) transform. This not only adds computational cost and complicates post-quantum security analysis but may also introduce side-channel risks. Adopting simpler IND-CPA secure KEMs (without the FO transform, as in Figure 1) could thus improve operational efficiency and reduce such risks. To ensure the reliability of our results, we performed experiments on both the x86-64 (Intel Core i7-1165G7 CPU) and ARM (Apple M4 chip, designed by Apple Inc., Cupertino, CA, USA; manufactured by TSMC, Hsinchu, Taiwan, China) architectures. We provide the complete source code and scripts for this work under the MIT License to ensure reproducibility and to support future research. Our public repository is https://github.com/CPA-TLS/CPA-TLS (accessed on 1 December 2025).

### 4.1. Performance Evaluation at the Hybrid Key Exchange Level

**Figure 10:**
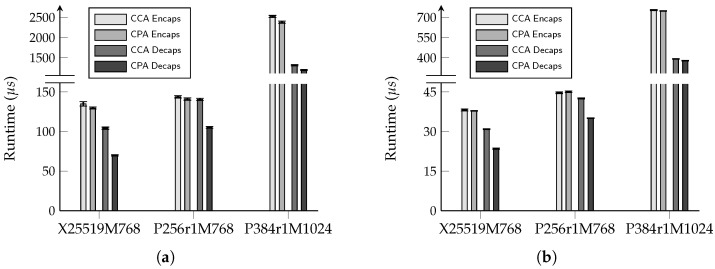
Performance comparison of hybrid key exchange. Bar heights show the mean, and error bars represent the 95% confidence interval. Labels “X25519M768”, “P256r1M768”, and “P384r1M1024” denote “X25519MLKEM768”, “SecP256r1MLKEM768”, and “SecP384r1MLKEM1024”, respectively. (**a**) Experimental environment: Ubuntu 24.04.3 TLS VM, Intel Core i7-1165G5 CPU, 16 GB RAM. (**b**) Experimental environment: Ubuntu 24.04.3 TLS VM, Apple M4 chip, 16 GB unified memory.

To quantify the performance improvement gained by replacing IND-CCA secure KEMs with IND-CPA secure ones, we modified the implementation of ML-KEM in the latest OpenSSL by removing the FO transform according to the method depicted in Figure 1, thereby obtaining its IND-CPA secure variant. We then used the openssl speed tool in a terminal to measure the computational cost of the three key exchange algorithms implemented in OpenSSL 3.6.0 (which correspond to those defined in the IETF draft [21]), using both the original IND-CCA secure ML-KEM implementation and our modified IND-CPA secure version. The experimental results are presented in Table 1, with a corresponding visualization shown in Figure 10. Each algorithm was executed for a default duration of 3 s, and the average time per operation was computed over 50 iterations. The raw data are available in Appendix A.

From the results, we observe that the FO transform primarily impacts the performance of the decapsulation algorithm. Consequently, the execution time of the encapsulation algorithm shows no significant change before and after the modification. In contrast, the decapsulation algorithm exhibits performance improvements of approximately 44.8%, 25.2%, and 9.1% on the Intel Core i7-1165G7 CPU, and 24.0%, 17.6%, and 3.5% on the Apple M4 chip, for X25519MLKEM768, SecP256r1MLKEM768, and SecP384r1MLKEM1024, respectively, as expected. The most substantial improvement for X25519MLKEM768 can be attributed to the fact that within the hybrid key exchange, the X25519 (EC)DH component is faster than SecP256r1 and SecP384r1. Thus, the optimization achieved by removing the FO transform has a more pronounced effect on the overall performance of X25519MLKEM768.

### 4.2. Performance Evaluation at the Protocol Level

For performance testing at the full TLS 1.3 protocol level, we simulated a client and server locally using two separate terminals to measure protocol efficiency, thereby excluding external interference such as network latency. We first launched one terminal, generated a certificate for the server, and then used the command

openssl s_server -cert server.crt -key server.key -accept 4443 -www
-groups <SELECTED_GROUPS>

to start a server listening on port 4443. The -groups parameter specifies the key exchange algorithm for the handshake, i.e., <SELECTED_GROUPS> is set to one of X25519MLKEM768, SecP256r1MLKEM768, or SecP384r1MLKEM1024. Another terminal runs the command

openssl s_time -connect localhost:4443 -new -time 3

to initiate TLS 1.3 handshakes and run this for 3 s, and we measure the total connections established across 50 repetitions of the experiment. Given that the removal of the FO transform mainly impacts the decapsulation algorithms in the client, we focus on evaluating the average user time per TLS 1.3 handshake connection with different hybrid key exchange algorithms.

To include the aforementioned three hybrid key exchange algorithms in OpenSSL’s default list, we modified the OpenSSL configuration file (openssl.cnf), specifying the supported groups in the [tls_system_default] section:

Groups = X25519MLKEM768:SecP256r1MLKEM768:SecP384r1MLKEM1024

**Figure 11:**
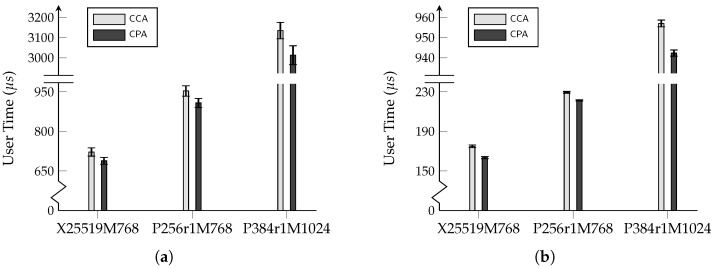
Performance comparison of TLS 1.3 handshake. Bar heights show the mean, and error bars represent the 95% confidence interval. Labels “X25519M768”, “P256r1M768”, and “P384r1M1024” denote “X25519MLKEM768”, “SecP256r1MLKEM768”, and “SecP384r1MLKEM1024”, respectively. (**a**) Experimental environment: Ubuntu 24.04.3 TLS VM, Intel Core i7-1165G5 CPU, 16 GB RAM. (**b**) Experimental environment: Ubuntu 24.04.3 TLS VM, Apple M4 chip, 16 GB unified memory.

Further details on this configuration can be found in the official OpenSSL documentation (https://openssl-library.org/post/2022-10-21-tls-groups-configuration/ accessed on 23 October 2025). The experimental results are presented in Table 2 and their corresponding visualization is in Figure 11. The raw data are available in Appendix B.

Compared to Table 1 and Figure 10, although removing the FO transform significantly improves the hybrid key exchange performance itself, the resulting efficiency gain at the overall protocol level is more modest, not exceeding 9% (considering the 95% confidence intervals). This is because the FO transform primarily impacts the runtime of KEM.Decaps, but during a single TLS connection, the client must perform one KEM.Gen, at least two digital signature verifications (for the certificate and the CertificateVerify signature), one MAC verification, and several key derivation operations, in addition to the KEM.Decaps. Consequently, while a positive performance gain is observed, employing a KEM with weaker security parameters yields a less significant efficiency improvement for the overall protocol than for the KEM operation itself.

Furthermore, it is important to note that removing the FO transform reduces the number of hash function invocations within the hybrid key exchange. As pointed out in Meta’s post-quantum transition update presented at the “Real World PQC Workshop” [23], the “extra hashing steps” used in transforms like FO can lead to “non-negligible capacity cost” when deploying post-quantum key exchange, which “can cost hundreds of thousands or even millions of dollars a year”. Scaling this to the global volume of TLS 1.3 traffic makes CPA-secure KEMs a more sustainable path for the post-quantum transition.

## 5. Conclusions

In this work, we have comprehensively demonstrated that CPA-secure KEMs are not only theoretically sufficient but also practically advantageous for the TLS 1.3 handshake in a hybrid key exchange setting. We bridged a critical gap between theoretical security models and practical deployment needs by providing the first formal security proofs, in both the ROM and QROM, that the security of the handshake can be reduced to the OW-CPA and IND-CPA security of a single component KEM within the hybrid construction.

Our security analysis is complemented by a rigorous performance evaluation. The experimental results substantiate a significant performance gain at the key exchange layer, with decapsulation speed improvements of up to 44.8%. While the overall protocol-level improvement is more modest, the elimination of the FO transform’s re-encryption step consistently enhances efficiency and, more importantly, reduces the codebase’s attack surface and potential operational costs.

This work firmly establishes CPA-secure KEMs as a viable, secure, and more efficient alternative for the ongoing standardization and deployment of post-quantum TLS 1.3. By relaxing the security requirement from IND-CCA to IND-CPA, we pave the way for simpler, faster, and potentially more secure implementations, thereby facilitating a smoother and more economical transition to the post-quantum era.

## Figures and Tables

**Table 1:** Performance comparison of hybrid key exchange. Values are expressed as the mean ± the margin of error of the 95% confidence interval.

Scheme	Encaps (μs)		Decaps (μs)	
	CCA	CPA	CCA	CPA
X25519MLKEM768 ^†^	134.82±2.81	129.60±1.25	104.16±1.43	69.93±0.79
SecP256r1MLKEM768 ^†^	143.67±1.45	140.98±1.50	140.48±1.41	105.14±1.15
SecP384r1MLKEM1024 ^†^	2523.1±25.25	2380.7±26.03	1317.1±15.77	1197.6±13.89
X25519MLKEM768 ^‡^	38.14±0.39	37.80±0.10	30.86±0.13	23.45±0.24
SecP256r1MLKEM768 ^‡^	44.65±0.29	45.04±0.31	42.53±0.18	35.04±0.08
SecP384r1MLKEM1024 ^‡^	753.96±3.34	747.47±2.47	392.21±2.39	378.29±2.36

^†^ Experimental environment: Ubuntu 24.04.3 LTS virtual machine, Intel Core i7-1165G7 CPU, 16 GB RAM. ^‡^ Experimental environment: Ubuntu 24.04.3 LTS virtual machine, Apple M4 chip, 16 GB unified memory.

**Table 2:** Performance comparison of TLS 1.3 handshake with different hybrid key exchange schemes (user time/connection). Values are the mean ± the margin of error of the 95% confidence interval.

Scheme	CCA (μs)	CPA (μs)
X25519MLKEM768 ^†^	721.81±15.74	688.28±13.04
SecP256r1MLKEM768 ^†^	953.15±19.64	907.63±16.96
SecP384r1MLKEM1024 ^†^	3134.9±40.47	3012.8±46.69
X25519MLKEM768 ^‡^	174.89±1.38	163.50±1.12
SecP256r1MLKEM768 ^‡^	229.46±0.88	221.32±0.61
SecP384r1MLKEM1024 ^‡^	957.00±1.69	942.33±1.54

^†^ Experimental environment: Ubuntu 24.04.3 LTS virtual machine, Intel Core i7-1165G7 CPU, 16 GB RAM. ^‡^ Experimental environment: Ubuntu 24.04.3 LTS virtual machine, Apple M4 chip, 16 GB unified memory.

## Data Availability

The original contributions presented in this study are included in this article. Further inquiries can be directed to the corresponding author.

## Appendix A. Raw Data for the Performance Evaluation at the Hybrid Key Exchange Level

We employed two different processor architectures for the hybrid key exchange level experiments: an Intel Core i7-1165G7 CPU (x86-64) and an Apple M4 chip (ARM).

### Appendix A.1. Raw Data on x86-64 Architecture at the Hybrid Key Exchange Level

The x86-64 benchmark configuration comprised an Ubuntu 24.04.3 LTS virtual machine equipped with an Intel Core i7-1165G7 CPU and 16 GB of RAM.

**Table A1:** Raw Data on x86-64 Architecture for the Performance Evaluation at the Hybrid Key Exchange Level for All 50 Runs (Time in μs).

Test Run	X25519MLKEM768				SecP256r1MLKEM768				SecP384r1MLKEM1024			
	Encaps		Decaps		Encaps		Decaps		Encaps		Decaps	
	CCA	CPA	CCA	CPA	CCA	CPA	CCA	CPA	CCA	CPA	CCA	CPA
Run 1	132.97	129.22	102.64	67.90	138.24	148.47	135.21	100.82	2508.80	2300.54	1452.72	1125.38
Run 2	129.98	128.49	103.44	65.98	143.38	131.18	143.91	102.38	2740.74	2434.64	1360.29	1201.61
Run 3	128.03	125.72	101.13	67.75	141.09	138.59	139.58	107.39	2420.54	2343.26	1318.92	1144.83
Run 4	123.21	132.96	99.64	72.53	143.85	136.75	152.96	102.91	2651.79	2338.58	1303.20	1192.48
Run 5	130.71	127.15	105.55	71.08	142.74	136.82	146.79	104.95	2656.53	2338.58	1267.01	1225.83
Run 6	127.43	122.78	99.34	68.08	139.21	143.28	138.73	105.79	2462.81	2397.43	1275.23	1184.89
Run 7	128.93	133.21	98.86	74.28	145.42	133.58	138.80	110.51	2571.18	2402.91	1220.12	1199.20
Run 8	121.85	134.09	98.47	69.61	143.40	139.66	135.75	102.19	2485.40	2468.93	1222.13	1227.35
Run 9	127.88	131.29	101.95	68.24	138.37	142.00	139.10	104.98	2305.32	2389.89	1295.25	1161.06
Run 10	132.95	121.41	102.41	62.55	146.58	141.48	138.75	98.07	2434.64	2458.75	1306.82	1195.65
Run 11	134.12	125.30	95.24	71.67	146.91	132.82	142.85	105.00	2429.98	2384.00	1282.38	1177.17
Run 12	127.55	130.12	102.69	68.12	142.10	146.72	141.82	101.01	2582.32	2365.08	1317.18	1162.25
Run 13	133.85	128.02	110.73	68.82	134.65	131.99	139.08	105.84	2466.78	2321.57	1308.17	1188.95
Run 14	133.19	122.61	103.18	70.95	143.28	138.97	140.29	103.62	2424.74	2448.30	1340.81	1169.29
Run 15	138.19	132.93	104.53	71.99	149.41	135.23	135.38	96.25	2531.75	2339.92	1328.58	1176.24
Run 16	127.71	125.89	100.59	68.79	143.48	142.25	142.16	107.27	2620.93	2232.21	1294.37	1247.90
Run 17	178.33	129.00	119.19	68.40	141.29	147.04	137.57	103.74	2500.00	2335.42	1230.39	1162.52
Run 18	165.86	123.60	97.68	71.09	137.44	141.32	136.42	97.74	2523.21	2355.73	1295.81	1127.51
Run 19	151.38	131.04	101.60	70.38	140.52	143.82	134.62	104.78	2627.80	2329.95	1300.35	1155.78
Run 20	127.54	133.47	104.21	65.78	141.34	137.85	145.96	106.21	2580.09	2321.57	1257.91	1251.57
Run 21	136.27	129.67	119.56	70.32	151.32	135.69	142.08	106.22	2434.43	2374.10	1289.91	1188.95
Run 22	144.48	136.41	103.54	71.40	137.64	147.75	136.70	104.86	2469.82	2244.74	1323.46	1266.47
Run 23	129.39	133.17	114.84	65.49	149.64	145.72	143.81	103.05	2542.52	2426.47	1298.47	1248.95
Run 24	132.91	125.38	105.82	76.37	146.44	132.35	143.59	101.04	2393.57	2315.46	1268.62	1157.57
Run 25	135.78	126.06	104.55	67.74	143.39	137.91	155.04	99.36	2508.42	2365.08	1316.94	1304.16
Run 26	136.55	123.66	101.94	65.70	140.51	142.30	139.80	95.80	2434.21	2306.50	1305.30	1210.53
Run 27	130.76	139.63	101.80	71.11	138.27	133.31	143.05	99.28	2516.95	2799.25	1199.84	1380.91
Run 28	133.34	129.17	106.33	66.60	154.04	130.81	129.99	104.00	2655.42	2284.62	1333.93	1231.85
Run 29	129.09	125.36	105.97	70.04	141.76	140.78	144.60	110.58	2609.46	2345.10	1333.93	1210.40
Run 30	139.33	131.29	103.23	67.68	140.05	136.65	147.17	106.78	2531.86	2355.73	1316.84	1211.38
Run 31	139.79	128.07	111.57	71.91	158.39	145.72	137.63	107.58	2580.09	2374.10	1231.40	1254.21
Run 32	154.05	134.08	101.95	73.12	145.73	139.26	140.31	108.70	2452.83	2616.33	1342.07	1224.83
Run 33	128.02	135.92	106.04	72.87	142.58	147.78	139.09	106.83	2475.17	2381.72	1423.78	1204.53
Run 34	128.28	132.77	102.46	73.57	139.88	142.35	142.99	111.38	2635.31	2374.50	1338.76	1163.42
Run 35	134.87	134.28	109.94	69.87	142.42	150.08	135.68	110.13	2722.27	2382.09	1371.38	1180.20
Run 36	134.76	131.79	103.51	71.71	151.86	149.82	138.32	112.38	2525.42	2381.72	1433.38	1142.20
Run 37	132.31	129.24	106.70	71.81	139.88	140.19	138.19	106.84	2523.36	2314.31	1325.35	1175.08
Run 38	140.11	129.81	107.56	68.64	137.58	139.78	131.59	105.44	2642.35	2416.87	1267.67	1226.26
Run 39	135.46	126.98	105.58	73.32	148.13	139.83	138.39	107.50	2477.06	2274.81	1447.43	1119.88
Run 40	132.62	127.79	105.98	74.99	144.80	150.79	143.56	105.03	2587.11	2456.72	1400.76	1228.36
Run 41	126.32	138.42	113.14	70.82	146.05	143.16	140.42	112.33	2479.20	2350.16	1324.44	1228.87
Run 42	136.61	135.77	102.24	67.93	140.24	141.69	136.70	105.42	2470.69	2428.69	1293.96	1150.58
Run 43	138.52	133.84	103.35	70.51	141.54	142.03	146.61	105.70	2557.94	2442.62	1318.00	1216.33
Run 44	127.88	121.98	102.24	66.02	147.63	139.32	136.29	111.23	2454.84	2462.69	1324.71	1132.29
Run 45	125.76	124.36	101.30	67.57	141.27	142.88	135.92	103.77	2469.03	2479.20	1396.54	1248.42
Run 46	136.16	130.88	98.56	68.45	143.36	146.90	140.63	103.61	2536.29	2283.52	1276.96	1213.85
Run 47	132.83	133.60	97.02	72.18	141.99	138.35	149.65	109.77	2575.89	2428.69	1356.16	1139.14
Run 48	134.26	126.67	101.31	72.91	159.25	141.11	143.69	111.63	2468.72	2315.46	1354.31	1201.29
Run 49	138.27	126.89	100.26	71.97	146.03	146.81	133.32	105.91	2510.57	2416.87	1343.01	1144.95
Run 50	134.34	128.77	106.51	69.93	139.27	148.06	143.43	103.57	2387.46	2297.61	1320.00	1197.27
**Mean**	134.82	129.60	104.16	69.93	143.67	140.98	140.48	105.14	2523.07	2380.69	1317.10	1197.61
**SD**	9.90	4.38	5.04	2.78	5.11	5.28	4.97	4.05	88.83	91.59	55.49	48.86
**95%CI(LB)**	132.00	128.35	102.73	69.14	142.22	139.48	139.07	103.99	2497.83	2354.66	1301.33	1183.73
**95%CI(UB)**	137.63	130.85	105.59	70.72	145.12	142.48	141.89	106.29	2548.32	2406.73	1332.87	1211.50

Experimental environment: Ubuntu 24.04.3 LTS virtual machine, Intel Core i7-1165G7 CPU, 16 GB RAM.

### Appendix A.2. Raw Data on ARM Architecture at the Hybrid Key Exchange Level

Benchmarking on the ARM platform was conducted using an Ubuntu 24.04.3 LTS virtual machine on an Apple M4 chip with 16 GB unified memory.

**Table A2:** Raw Data on ARM Architecture for the Performance Evaluation at the Hybrid Key Exchange Level for All 50 Runs (Time in μs).

Test Run	X25519MLKEM768				SecP256r1MLKEM768				SecP384r1MLKEM1024			
	Encaps		Decaps		Encaps		Decaps		Encaps		Decaps	
	CCA	CPA	CCA	CPA	CCA	CPA	CCA	CPA	CCA	CPA	CCA	CPA
Run 1	37.37	36.93	30.49	22.59	44.33	44.31	42.26	34.86	733.38	747.94	381.58	383.58
Run 2	37.75	36.87	30.57	22.70	44.40	44.25	42.27	34.74	744.63	734.57	388.96	370.83
Run 3	37.63	38.26	30.51	22.77	44.29	44.09	42.31	34.48	742.77	736.56	385.46	372.25
Run 4	37.73	37.33	30.66	25.78	44.29	44.36	42.20	34.66	742.67	736.45	387.35	371.61
Run 5	37.74	37.33	30.80	23.01	44.39	44.47	42.30	34.84	774.21	741.29	405.42	373.00
Run 6	37.98	37.59	30.63	23.32	44.32	44.65	42.28	34.85	742.86	761.04	390.08	389.78
Run 7	37.79	38.58	30.63	23.01	44.40	44.83	42.29	34.68	755.35	739.37	390.06	373.79
Run 8	37.76	37.46	30.74	23.09	44.31	44.65	42.42	34.75	744.42	749.75	386.50	375.33
Run 9	37.89	37.74	30.78	23.18	44.39	44.53	42.24	34.73	752.53	741.66	392.97	374.58
Run 10	38.08	37.67	31.01	23.11	44.16	44.73	42.34	34.87	768.25	745.53	401.67	375.42
Run 11	39.11	38.10	30.80	23.17	44.33	44.55	42.47	35.13	742.94	744.97	385.65	374.72
Run 12	37.96	37.48	30.67	23.10	46.53	44.69	42.47	35.05	765.29	738.09	405.97	373.18
Run 13	38.08	37.57	30.64	23.15	44.37	47.02	42.28	34.77	748.32	743.68	393.80	376.36
Run 14	37.79	37.73	30.72	23.05	44.42	45.22	42.27	34.87	755.67	751.90	390.98	378.83
Run 15	37.85	37.96	30.87	26.87	44.39	45.11	42.66	35.11	748.69	777.72	387.90	394.89
Run 16	37.98	37.76	32.63	23.34	44.59	44.78	42.74	34.80	748.13	753.58	385.70	381.68
Run 17	38.02	37.51	30.80	26.70	44.28	44.47	42.33	35.07	771.01	740.74	408.64	375.58
Run 18	37.84	39.09	30.83	23.14	44.96	44.33	42.73	34.86	768.24	745.45	405.90	375.72
Run 19	38.00	37.59	30.70	23.17	44.57	44.60	42.52	34.92	749.25	761.01	389.02	382.30
Run 20	37.97	37.92	30.72	23.21	44.49	44.58	42.47	35.08	753.90	753.58	388.97	374.64
Run 21	37.88	37.75	30.71	23.22	44.50	44.64	42.35	34.84	794.07	744.79	418.24	374.26
Run 22	37.98	37.92	30.68	23.18	44.44	46.93	42.34	35.41	752.53	741.75	390.27	376.03
Run 23	37.96	37.66	30.81	23.26	44.29	44.99	42.36	35.25	754.15	738.09	394.89	373.32
Run 24	37.97	37.85	30.78	23.19	44.25	44.91	42.29	34.89	749.00	761.42	387.95	390.88
Run 25	37.91	37.76	30.80	23.26	44.24	45.47	42.46	35.12	778.61	754.53	404.55	385.85
Run 26	37.91	37.77	30.80	24.89	44.41	45.17	42.43	35.01	745.90	765.89	387.15	392.05
Run 27	37.90	37.76	30.81	23.22	44.43	51.36	42.32	35.51	748.13	742.39	386.90	373.83
Run 28	37.70	37.68	30.94	23.14	44.38	45.34	42.33	35.21	771.02	751.69	406.23	375.75
Run 29	37.82	37.71	33.25	23.19	44.62	44.91	43.08	35.17	741.20	745.16	385.95	376.03
Run 30	37.78	37.68	30.80	23.30	48.10	46.21	44.68	36.03	754.00	743.68	386.35	374.81
Run 31	37.89	37.78	30.84	23.23	44.52	45.69	42.50	35.48	752.45	744.42	389.12	374.81
Run 32	37.71	37.86	30.80	23.27	50.35	44.92	42.47	35.41	751.69	764.53	389.32	389.53
Run 33	47.16	37.81	30.81	23.22	44.42	45.35	42.38	35.40	751.31	743.13	388.16	372.86
Run 34	40.13	37.88	30.85	23.21	44.77	45.04	42.25	35.22	770.64	746.08	393.34	375.70
Run 35	37.74	37.90	30.87	23.17	44.20	44.99	46.11	35.28	744.60	744.79	386.60	374.36
Run 36	37.85	37.73	30.79	23.21	44.38	45.25	42.34	35.38	742.30	748.06	386.85	417.65
Run 37	38.02	37.81	30.83	23.23	44.37	45.09	42.36	35.33	752.26	764.31	390.78	393.34
Run 38	37.94	37.84	30.72	23.27	44.54	45.11	42.42	35.38	767.84	741.94	414.47	375.70
Run 39	38.07	37.70	30.78	24.78	44.35	44.74	42.52	35.06	755.05	742.39	392.98	376.06
Run 40	37.94	38.56	30.77	23.30	44.49	44.79	42.41	35.45	772.01	744.97	403.03	375.89
Run 41	38.03	38.23	30.87	23.32	44.28	44.59	42.39	35.06	753.86	750.56	392.29	375.80
Run 42	38.15	37.95	30.80	23.31	44.27	44.80	42.46	34.92	760.04	743.68	388.90	374.67
Run 43	37.93	37.90	30.85	23.27	44.27	44.61	42.41	34.87	756.58	740.56	391.62	372.62
Run 44	37.91	38.00	30.84	23.29	44.58	44.86	42.52	34.85	747.50	744.60	386.95	374.98
Run 45	37.88	37.86	30.60	23.29	44.61	44.45	42.40	34.87	750.63	742.39	389.54	374.30
Run 46	37.92	37.76	30.95	23.35	44.40	44.64	42.34	34.92	748.26	744.05	384.76	374.53
Run 47	37.98	37.75	30.83	23.36	44.36	44.60	42.42	34.87	746.08	747.01	385.60	376.18
Run 48	37.89	37.95	30.80	23.33	44.44	44.73	42.36	35.05	744.68	742.21	383.58	373.00
Run 49	37.81	37.70	30.76	23.30	44.52	44.94	42.37	34.83	743.68	747.31	384.57	377.00
Run 50	37.78	37.87	30.96	23.55	44.45	44.59	42.47	35.03	745.26	752.26	386.80	374.36
**Mean**	38.14	37.80	30.86	23.45	44.65	45.04	42.53	35.04	753.96	747.47	392.21	378.29
**SD**	1.36	0.35	0.45	0.85	1.03	1.08	0.63	0.28	11.74	8.70	8.42	8.29
**95%CI(LB)**	37.75	37.70	30.73	23.21	44.36	44.73	42.35	34.96	750.62	745.00	389.81	375.93
**95%CI(UB)**	38.52	37.90	30.98	23.70	44.94	45.34	42.71	35.12	757.29	749.94	394.60	380.64

Experimental environment: Ubuntu 24.04.3 LTS virtual machine, Apple M4 chip, 16 GB unified memory.

## Appendix B. Raw Data for the Performance Evaluation at the Protocol Level

We employed two different processor architectures for the protocol level experiments: an Intel Core i7-1165G7 CPU (x86-64) and an Apple M4 chip (ARM).

### Appendix B.1. Raw Data on x86-64 Architecture at the Protocol Level

The x86-64 benchmark configuration comprised an Ubuntu 24.04.3 LTS virtual machine equipped with an Intel Core i7-1165G7 CPU and 16 GB of RAM.

**Table A3:** Raw Data on x86-64 Architecture for the Performance Evaluation at the Protocol Level for All 50 Runs (Time in μs).

Test Run	X25519MLKEM768		SecP256r1MLKEM768		SecP384r1MLKEM1024	
	CCA	CPA	CCA	CPA	CCA	CPA
Run 1	620.44	695.42	862.07	915.49	3071.16	2901.23
Run 2	724.07	667.24	928.14	1000.00	3311.04	2712.93
Run 3	642.86	663.79	844.77	993.38	2811.50	3065.02
Run 4	681.62	668.95	902.56	1016.22	3049.18	3031.25
Run 5	734.30	638.84	966.08	884.04	2924.53	2912.62
Run 6	796.81	694.09	910.93	924.17	3244.15	3000.00
Run 7	689.66	705.47	939.73	900.11	3288.14	2959.50
Run 8	748.82	728.42	906.49	866.94	3234.32	2904.19
Run 9	727.45	700.35	961.74	1005.08	3081.97	2915.36
Run 10	691.36	650.62	925.34	935.55	3092.11	2896.34
Run 11	679.86	680.27	1078.84	859.21	3235.29	3047.62
Run 12	658.98	604.44	996.92	925.53	3407.64	2878.79
Run 13	794.39	591.13	1120.19	903.36	3181.82	2984.62
Run 14	893.97	639.00	1079.06	909.09	3168.32	3015.38
Run 15	825.29	681.62	884.21	830.04	3414.63	2943.04
Run 16	713.06	644.10	924.09	929.07	3277.59	3250.77
Run 17	687.83	758.75	1006.86	863.16	3074.43	3049.18
Run 18	790.66	615.64	921.61	845.62	3037.97	3177.57
Run 19	758.62	709.52	921.18	868.20	3019.48	3134.80
Run 20	716.85	692.90	936.90	926.32	3215.43	2690.06
Run 21	703.13	698.47	1029.72	989.01	3192.18	2943.04
Run 22	717.45	681.82	961.12	939.81	3067.09	2946.71
Run 23	713.06	712.42	917.62	905.22	3121.02	2893.89
Run 24	700.09	712.45	980.60	884.15	3112.58	3203.88
Run 25	663.13	671.77	1082.73	922.92	3040.75	3015.87
Run 26	686.81	687.29	1053.20	836.73	2981.37	2984.13
Run 27	728.97	700.98	927.42	1093.17	3290.32	2944.98
Run 28	782.18	652.95	961.34	896.09	2664.58	3278.15
Run 29	731.93	666.67	900.72	913.87	3265.99	3476.82
Run 30	750.45	683.01	848.26	881.76	3069.31	3044.87
Run 31	659.63	630.25	987.78	849.34	3164.56	3149.35
Run 32	740.74	703.05	994.59	938.82	3233.33	2830.77
Run 33	682.01	769.23	885.42	892.12	3202.61	2943.04
Run 34	664.24	631.23	926.83	898.99	3094.46	2919.25
Run 35	774.25	707.16	989.43	810.81	3071.90	2974.68
Run 36	831.68	660.87	1002.18	832.54	3225.81	3535.35
Run 37	779.82	750.72	856.55	933.63	3084.42	2971.25
Run 38	670.84	651.24	904.11	789.20	3344.26	3110.37
Run 39	754.04	723.51	931.22	826.99	3169.93	3187.92
Run 40	634.63	721.75	1094.09	907.08	3003.10	3218.39
Run 41	783.37	666.09	1011.24	898.64	3197.49	2871.29
Run 42	745.50	818.60	878.50	853.91	2852.76	2839.12
Run 43	730.24	744.39	879.85	856.24	3202.61	3034.06
Run 44	701.28	669.49	913.65	962.80	3123.03	3079.37
Run 45	663.13	649.02	884.35	885.31	3130.99	2821.32
Run 46	743.06	722.37	962.73	910.99	3190.79	3127.04
Run 47	735.03	784.12	1081.38	847.64	3193.55	2980.77
Run 48	641.57	676.03	953.39	997.85	2993.63	2774.39
Run 49	696.20	675.79	939.92	976.95	3072.10	3049.18
Run 50	705.13	760.87	899.62	948.48	3247.59	3019.48
**Mean**	721.81	688.28	953.15	907.63	3134.90	3012.78
**SD**	55.39	45.88	69.11	59.67	142.41	164.29
**95% CI**	[706.07, 737.55]	[675.24, 701.32]	[933.51, 972.79]	[890.67, 924.59]	[3094.42, 3175.37]	[2966.09, 3059.47]

Experimental environment: Ubuntu 24.04.3 LTS virtual machine, Intel Core i7-1165G7 CPU, 16 GB RAM.

### Appendix B.2. Raw Data on ARM Architecture at the Protocol Level

Benchmarking on the ARM platform was conducted using an Ubuntu 24.04.3 LTS virtual machine on an Apple M4 chip with 16 GB unified memory.

**Table A4:** Raw Data on ARM Architecture for the Performance Evaluation at the Protocol Level for All 50 Runs (Time in μs).

Test Run	X25519MLKEM768		SecP256r1MLKEM768		SecP384r1MLKEM1024	
	CCA	CPA	CCA	CPA	CCA	CPA
Run 1	173.36	160.78	229.30	226.85	963.42	952.38
Run 2	166.28	161.37	230.18	219.74	960.80	939.12
Run 3	167.53	156.82	227.05	224.79	960.80	944.99
Run 4	173.35	160.83	226.87	223.04	962.64	940.45
Run 5	163.47	157.48	226.75	223.35	972.97	946.07
Run 6	165.54	157.06	228.25	221.60	955.14	942.06
Run 7	171.28	159.91	227.35	221.96	974.53	933.08
Run 8	173.06	157.44	230.80	223.23	963.33	933.08
Run 9	167.39	160.58	226.95	218.41	956.94	944.29
Run 10	172.22	160.97	231.76	222.29	953.74	948.32
Run 11	171.34	160.12	229.37	216.66	959.89	944.11
Run 12	166.69	161.30	233.33	218.14	950.79	933.96
Run 13	170.95	163.44	240.28	225.73	960.34	940.29
Run 14	169.35	161.44	237.58	223.09	959.89	942.51
Run 15	173.96	162.74	232.77	222.30	955.11	947.22
Run 16	176.52	163.73	233.66	219.21	956.94	945.18
Run 17	170.48	161.37	230.65	221.11	959.23	939.57
Run 18	177.52	161.24	229.92	220.17	951.24	955.08
Run 19	174.51	158.68	230.19	221.67	954.20	949.46
Run 20	173.87	162.33	232.94	220.76	952.38	937.79
Run 21	180.98	163.47	229.83	222.08	950.79	946.07
Run 22	173.38	175.75	226.82	223.18	956.02	946.52
Run 23	176.85	162.64	227.83	218.19	951.24	933.52
Run 24	177.90	163.10	227.37	219.70	953.29	940.01
Run 25	174.97	177.02	227.65	224.84	953.29	952.16
Run 26	172.93	164.52	231.99	219.63	960.80	934.84
Run 27	182.24	162.78	227.72	218.92	954.20	947.67
Run 28	171.00	161.53	230.91	221.40	959.89	945.63
Run 29	174.89	166.49	228.53	217.91	953.74	947.42
Run 30	178.70	160.64	226.42	221.57	958.06	938.68
Run 31	173.19	165.11	226.09	222.03	959.89	934.40
Run 32	177.56	164.95	228.43	219.87	947.62	940.29
Run 33	182.24	163.83	224.87	220.05	949.43	936.03
Run 34	173.87	165.69	225.05	222.95	959.89	944.73
Run 35	173.74	164.24	225.67	218.67	951.93	938.24
Run 36	183.02	163.56	227.78	221.23	954.20	937.79
Run 37	173.91	164.75	228.75	222.15	949.88	942.06
Run 38	175.87	165.52	228.55	222.42	953.74	941.62
Run 39	177.21	171.36	228.82	223.06	964.03	948.56
Run 40	175.38	166.69	230.30	222.63	957.40	940.29
Run 41	178.06	164.91	230.01	223.11	957.40	942.51
Run 42	175.78	167.59	230.47	222.49	960.34	951.25
Run 43	182.27	165.35	226.92	221.22	943.76	942.51
Run 44	180.83	165.01	228.25	217.89	961.72	946.77
Run 45	178.69	164.14	229.43	221.19	951.93	945.18
Run 46	182.87	167.94	227.87	220.44	952.38	939.12
Run 47	182.96	163.34	233.33	218.28	958.51	934.40
Run 48	176.13	163.66	226.67	222.49	958.77	938.24
Run 49	177.75	163.76	228.96	221.81	952.61	939.12
Run 50	180.51	166.03	235.72	220.68	968.82	942.06
**Mean**	174.89	163.50	229.46	221.32	957.00	942.33
**SD**	4.85	3.93	3.08	2.15	5.93	5.43
**95% CI**	[173.51, 176.26]	[162.38, 164.62]	[228.58, 230.34]	[220.71, 221.93]	[955.31, 958.68]	[940.79, 943.88]

Experimental environment: Ubuntu 24.04.3 LTS virtual machine, Apple M4 chip, 16 GB unified memory.
